# Comparative Review of Microglia and Monocytes in CNS Phagocytosis

**DOI:** 10.3390/cells10102555

**Published:** 2021-09-27

**Authors:** Megumi Andoh, Ryuta Koyama

**Affiliations:** 1 Laboratory of Chemical Pharmacology, Graduate School of Pharmaceutical Sciences, The University of Tokyo, Tokyo 113-0033, Japan; 21100750megumi@gmail.com; 2 Institute for AI and Beyond, The University of Tokyo, Tokyo 113-0033, Japan

**Keywords:** microglia, macrophage, monocyte, phagocytosis, infiltration

## Abstract

Macrophages maintain tissue homeostasis by phagocytosing and removing unwanted materials such as dead cells and cell debris. Microglia, the resident macrophages of the central nervous system (CNS), are no exception. In addition, a series of recent studies have shown that microglia phagocytose the neuronal synapses that form the basis of neural circuit function. This discovery has spurred many neuroscientists to study microglia. Importantly, in the CNS parenchyma, not only microglia but also blood-derived monocytes, which essentially differentiate into macrophages after infiltration, exert phagocytic ability, making the study of phagocytosis in the CNS even more interesting and complex. In particular, in the diseased brain, the phagocytosis of tissue-damaging substances, such as myelin debris in multiple sclerosis (MS), has been shown to be carried out by both microglia and blood-derived monocytes. However, it remains largely unclear why blood-derived monocytes need to invade the parenchyma, where microglia are already abundant, to assist in phagocytosis. We will also discuss whether this phagocytosis can affect the fate of the phagocytosing cell itself as well as the substance being phagocytosed and the surrounding environment in addition to future research directions. In this review, we will introduce recent studies to answer a question that often arises when studying microglial phagocytosis: under what circumstances and to what extent blood-derived monocytes infiltrate the CNS and contribute to phagocytosis. In addition, the readers will learn how recent studies have experimentally distinguished between microglia and infiltrating monocytes. Finally, we aim to contribute to the progress of phagocytosis research by discussing the effects of phagocytosis on phagocytic cells.

## 1. Introduction

The CNS, consisting of the brain and the spinal cord, is separated from the periphery by the presence of the blood–brain barrier (BBB), meninges, and the choroid plexus [1,2,3]. Since the major immune cells present in the parenchyma are microglia [4,5], unwanted materials (dead cells, aggregated proteins, etc.) in the parenchyma are removed mainly by microglial phagocytosis under physiological conditions [6,7]. Although not discussed in this review, astrocytes, another type of glial cell, also have the ability to phagocytose [8].

Dead cells and aggregated proteins that remain unremoved in the parenchyma cause tissue damage via the release of reactive oxygen species and various inflammatory mediators [9,10,11,12]. Thus, the phagocytosis of this debris by microglia has been thought to be important for homeostasis in the brain and spinal cord. On the other hand, in diseases with physical damage or excessive inflammatory responses, blood-derived monocytes infiltrate the parenchyma due to a loosening or disruption of the BBB [7,13,14]. These monocytes infiltrating the parenchyma have been shown to exert phagocytic activity in various diseases [7,13]. For example, in addition to myelin debris in MS [15] and apoptotic neurons in stroke [16], blood-derived monocytes have been suggested to phagocytose amyloid-beta (Aβ) and synapses [17,18]. Because microglia and blood-derived monocytes have different cell lineages and gene expression [6,19,20], differences in phagocytic capacity are also expected. However, many studies have analyzed microglia and blood-derived monocytes together as phagocytes without distinguishing between them or have examined each cell under different conditions, and little has been done to examine the differences in phagocytic capacity between microglia and blood-derived monocytes under the same conditions.

In recent years, thanks to technical advances in cell lineage analysis and genetic analysis, several methods have been developed to distinguish between microglia and blood-derived monocytes and to manipulate them separately, making it possible to directly compare their phagocytic abilities [21,22]. The heterogeneity of phagocytosis by cell type offers the possibility of manipulating phagocytosis in a cell-type-specific manner and is expected to be a therapeutic target for various diseases in which phagocytosis plays an essential role. In this review, we first introduce the phagocytosis by microglia in the physiological conditions. Next, we focus on studies that examined the phagocytosis of microglia and blood-derived monocytes under conditions in which blood-derived monocytes invade the parenchyma. We will then discuss the extent to which the phagocytic capacity of the two types of macrophages differs and what is responsible for this difference. Specifically, we will mention how many monocytes infiltrate the parenchyma in each disease and how long they stay there and focus on factors that may affect phagocytosis, such as cytokines.

**Figure 1:**
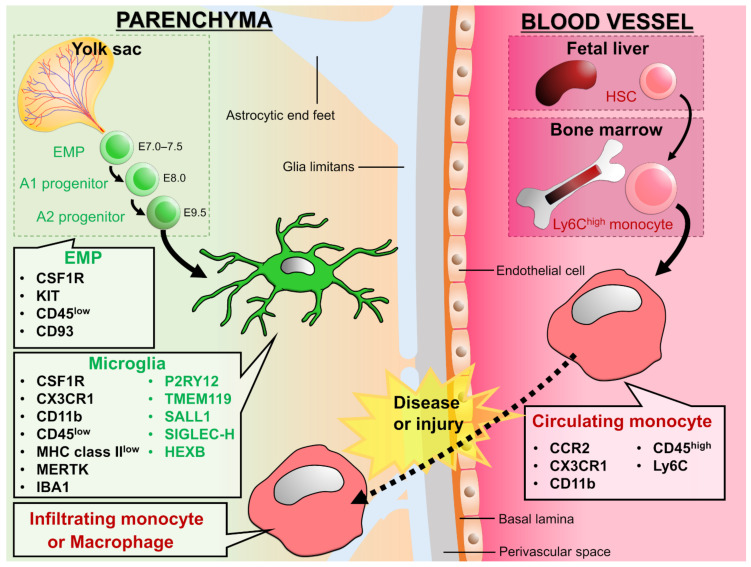
Development and expression signature of microglia and circulating monocytes: Erythro-myeloid progenitors (EMPs) from yolk sac invade into the CNS parenchyma during embryonic stage and differentiate into microglia. On the other hand, hematopoietic stem cells (HSCs) from fetal liver or bone marrow differentiate into monocytes and circulate in the blood vessels. In the physiological conditions, microglia are a main macrophage in the CNS parenchyma, but some diseases and injuries induce infiltration of circulating monocytes into the CNS parenchyma. We can distinguish between microglia and infiltrating monocytes by the genes listed in the figure. The molecules shown in green are called homeostatic microglial markers and are often considered as microglia specific. However, TMEM119, P2Y12, SIGLEC-H, and SALL1 are down-regulated in microglia in diseased brains [33,47,48], and peripheral macrophages begin to express molecules such as TMEM119, P2Y12, and SALL1 after infiltrating the brain parenchyma [49]. Therefore, it would be difficult to distinguish microglia from monocytes by these markers in situations where peripheral monocytes are infiltrating. On the other hand, HEXB has been suggested to be microglia-specific in both physiological and pathological conditions [50]. For signatures on other types of macrophages, please refer to the reviews by Prinz et al. and Li et al. [6,19].

Although circulating monocytes basically differentiate into macrophages and dendritic cells after infiltrating into tissues, the possibility that they exert their phagocytic function even before differentiating into macrophages cannot be excluded. Therefore, in this review, for studies in which differentiation into macrophages cannot be clearly confirmed, monocytes after invasion will be described as blood-derived monocytes but not as macrophages. Although microglia and blood-derived monocytes show similar characteristics in the parenchyma, the methods and criteria used to distinguish between them (degree of expression of molecular markers and morphology) will be introduced and summarized in Table 1 and Table 2. In addition, please refer to Figure 1, which shows each type of macrophages.

Many studies have focused on the removal of unwanted materials by phagocytes and the effects of their disruption on the surrounding environment and cell functions. At the same time, the significance of phagocytosis in phagocytes themselves, such as the functional changes that occur after phagocytosis, has also been examined. Studies from this perspective may reveal that phagocytosis has important biological implications beyond the mere clearance of unwanted substances. Furthermore, examining changes in both phagocytosing and phagocytosed cells may provide important insights into phagocytosis as a therapeutic target. Therefore, in the last part of this review, we will discuss why phagocytes phagocytose and the significance of this process.

## 2. Phagocytosis in the Intact Brain

Microglia have been reported to be involved in the development and progression of neurodegenerative diseases by phagocytosing apoptotic cells, aggregated proteins, and invading pathogens in the brain [7,10]. However, in the last decades or so, it has been revealed that microglia exert their phagocytic ability not only in diseased brains but also in healthy brains [7,10]. Further, the failure of phagocytosis by microglia under the physiological conditions could lead to disruption of neural circuit homeostasis and pathogenesis [7,10]. In this chapter, we outline the phagocytosis by microglia in the intact brain, where there are few infiltrations of blood-derived monocytes. This information will help our understanding of the role of microglial phagocytosis in the diseased brain.

Neuronal apoptosis and the removal of dead cell debris by microglia occur in the brain throughout the lifetime of an individual [51,52,53,54,55,56]. Phagocytosis of apoptotic neurons by microglia has been validated by visualization of microglia and apoptotic markers such as condensed nuclei, phosphatidylserine, and Cleaved Caspase-3. Microglia uptake apoptotic neurons with phagocytic cups, or pouches, during development [57,58] and adulthood [56]. More recently, it has been shown that microglia phagocytose apoptotic oligodendrocytes in the cortical white matter during development, which is the initiation period of myelin formation [59]. Microglia also phagocytose myelin debris in the cortex during aging and under the physiological condition where demyelination and remyelination constantly occur [60,61].

Microglia phagocytose not only apoptotic cells but also live cells. In the subventricular zone, a neurogenic niche, microglia have been observed to surround, uptake, and digest neural progenitor cells (NPCs) [51]. In addition, the number of NPCs in the cerebral cortex increased when microglia were removed by clodronate administrated into the ventricle of embryonic rats, indicating that phagocytosis of NPCs regulates neuronal density. Actually, it has been confirmed that the number of neurons in the prefrontal cortex of autistic patients is increased compared to control patients [62], so it is possible that deficient removal of NPCs causes the increase in neuronal density and abnormal wiring of neural circuits.

Among phagocytosis in the healthy brain, synaptic phagocytosis has been most intensively studied. Microglial ability to reorganize neuronal circuits through synaptic phagocytosis is an attractive research target for neuroscientists. In the developing brain, excess synapses are formed and then decrease, and the density remains constant during adulthood. Microglial phagocytosis is involved in this developmental synaptic decrease [63,64,65]. Furthermore, it has been shown that microglia contribute to the formation of functional neural circuits by phagocytosing synapses with weak neural activity and allowing synapses with strong neural activity to remain [64]. Indeed, inhibition of synaptic phagocytosis by microglia alters synaptic density, leading to the development of autism and epilepsy [66,67,68], and synaptic phagocytosis by microglia is defective in animal models of autism [69]. In addition, the synaptic phagocytosis by microglia is likely to be involved in synaptic plasticity associated with sensory stimulation and learning [70,71]. For more details on the molecular mechanisms of synaptic phagocytosis by microglia, please refer to the review published by Andoh et al. [72].

## 3. Phagocytosis by Microglia and Infiltrating Monocytes in the Diseased Brain

The immune response in the brain parenchyma of naïve adult mice is carried out mainly by microglia, which account for approximately 98% of immune cells (CD45 positive (CD45^+^)) in the brain [5]. Therefore, in the diseased brain, where circulating immune cells can infiltrate the parenchyma, both microglia and blood-derived monocytes can exert their phagocytic abilities simultaneously or complementarily in the same environment. Therefore, in Chapter 3, we will discuss the different targets of phagocytosis in the diseased brain: myelin debris, apoptotic cells, and tumor cells. Methods and criteria used to distinguish between microglia and blood-derived monocytes are introduced in the text and Table 1 and Table 2. We will also mention the advantages and limitations of those methods in the text and tables.

### 3.1. Myelin

In diseases with demyelination, such as MS, it has been suggested that myelin debris is not removed and instead accumulates in the brain parenchyma after demyelination, promotes inflammatory responses, and inhibits oligodendrocyte differentiation as well as myelin formation [73,74,75]. Therefore, it has been suggested that the clearance of myelin debris by phagocytes could be a therapeutic target for these diseases. In particular, since circulating monocytes are known to infiltrate the parenchyma in MS, several studies have examined whether microglia or macrophages phagocytose more myelin debris.

In vitro research has suggested that microglia have a higher capacity of myelin phagocytosis compared to macrophages at a single cell level. Mosley et al. used microglia and macrophages (collected by peritoneal perfusion, no molecular marker information) isolated from rats [76]. When the same amount of myelin was added, the amount of myelin phagocytosed was higher in microglia. The amount of myelin uptake by microglia monotonically increased until 18 h after myelin addition, whereas the amount of myelin phagocytosed by macrophages decreased after 6 h. Another study compared myelin phagocytosis by microglia isolated from the human surgical brain and by human blood-derived macrophages in vitro (monocytes differentiated by Granulocyte-Macrophage Colony-Stimulating Factor and Macrophage Colony-Stimulating Factor (M-CSF) treatment) [77]. The percentage of cells that incorporated the myelin marker, myelin binding protein (MBP), was higher in microglia than in macrophages.

Which phagocytoses more myelin debris at the individual level (i.e., per animal), microglia or blood-derived monocytes? In vitro studies are useful for directly comparing myelin phagocytosis by microglia and macrophages, but the results are difficult to interpret because they do not reflect the situation in the actual pathology (e.g., the ratio of infiltrating macrophages to microglia and the distance between each cell and myelin). In vivo validation has been performed mainly in the spinal cord; Kucharova et al. tested whether microglia or macrophages phagocytose more myelin in vivo [28]. By transplanting bone-marrow-derived macrophages expressing beta-actin-EGFP (Iba1^+^ and F4/80^+^) into mice with spinal cord injury, the authors were able to distinguish between microglia and infiltrating macrophages and calculated the percentage of cells that phagocytosed MBP. The results showed that 1 week after spinal cord injury, the cell density was 111 ± 14.4 (/0.1 mm^2^) for macrophages and 11.63 ± 2 (/0.1 mm^2^) for microglia. The percentage of cells that phagocytosed MBP (% cells containing phagocytosed MBP) was 27 ± 3% for macrophages and 45 ± 7% for microglia. Since the multiplication of these values was greater in macrophages, the above results suggest that macrophages have a higher myelin phagocytosis capacity as a whole.

A similar trend has been observed in rats; Rinner et al. compared the responsiveness of microglia and bone-marrow-derived macrophages in a rat model of experimental autoimmune encephalitis (EAE) through the injection of MBP-responsive T cells [36]. Using macrophage removal by irradiation, the authors distinguished resident microglia from infiltrating macrophages. 5 days after EAE induction, in the spinal cord, the cell density of macrophages was several times higher than that of microglia. The percentage of myelin uptake by each cell was approximately 5% in infiltrating macrophages and approximately 30% in microglia. These results suggest that microglia have a higher myelin phagocytosis capacity per cell. For phagocytes, spatial location with myelin is also an important factor for efficient myelin phagocytosis. Yamasaki et al. examined the involvement of microglia and monocyte-derived macrophages in the pathogenesis of EAE, focusing on the distance between phagocytes and myelin debris [15]. They used CCR2-RFP::CX3CR1-GFP mice, in which microglia and macrophages were labeled with GFP and RFP, respectively. The interaction between myelin and each phagocyte was quantified by using serial block-face scanning electron microscopy. A comparative analysis of microglia and macrophages located near spinal cord axons showed that the percentage of cells that phagocytosed myelin was higher in macrophages. In addition, the authors found that macrophages contacted almost all of the axons that microglia were in contact with. Again, it seems that microglia phagocytose more myelin at a single cell level, while macrophages phagocytose myelin phagocytosis in the whole individual.

The temporal window of phagocytic capacity was also compared between microglia and infiltrating macrophages after spinal cord injury [30]. In this study, lysozyme M EGFP-knock-in mice were used, in which only bone-marrow-derived monocytes expressed GFP, allowing to distinguish between microglia and macrophages. 1 day after injury, the periaxonal phagocytes were mostly microglia, while 3 and 7 days after injury, they were mostly macrophages. The phagocytic capacity of periaxonal microglia and macrophages was examined by analyzing the percentage of cells that incorporated autofluorescence into the cells: Microglia reached a peak at 3 days after injury and then rapidly approached zero, whereas nearly half of macrophages retained autofluorescence until 42 days after injury. Furthermore, the phagocytosis of myelin debris in vitro resulted in more cell death in macrophages after myelin phagocytosis. The promoted cell death in infiltrating macrophages was also confirmed in vivo. These results indicate that microglia can efficiently phagocytose and digest myelin in the early stage of injury and prevent their own cell death. On the other hand, infiltrating macrophages may continue to phagocytose myelin without being able to digest it, thereby making themselves susceptible to cell death. It has been suggested that microglia are involved in the death of macrophages in the demyelinating region. Plemel et al. showed that infiltrating macrophages did not proliferate, and their density decreased with time after EAE induction [37]. In the demyelinating region, microglia were activated (promoted proliferation, increased CD45 expression, and decreased expression levels of homeostatic markers such as CX3CR1) and surrounded macrophages. Furthermore, microglial depletion inhibited the macrophage reduction and promoted axonal loss. From these results, the authors discussed that microglia may act in a neuroprotective manner by suppressing the excessive inflammatory response via removal of infiltrating macrophages.

The above findings suggested that microglia tended to have higher myelin phagocytic capacity in vitro, while macrophages tended to have higher myelin phagocytic capacity in vivo. This difference may be due to the divergence in experimental methods, but it is important to consider all of these factors to determine the direction of future research. For example, although microglia have a higher phagocytic capacity per cell, macrophages efficiently phagocytose myelin in vivo due to their distance from the axon and persistence of phagocytotic ability, so the promotion of microglial migration to the axon may be effective in the removal of myelin debris.

Surrounding inflammatory mediators in the extracellular milieu also induce differences in the myelin phagocytic capacity of microglia and macrophages [78]. In MS, increased expression of interferon-γ (IFN-γ), tumor necrosis factor-α (TNF-α), and interleukin-4 (IL-4), and decreased expression of interleukin-10 (IL-10) have been confirmed [79,80]. Among these inflammatory mediators, TNF-α promotes myelin phagocytosis by microglia but does not affect myelin phagocytosis by macrophages [80]. In addition, IFN-γ inhibits myelin phagocytosis by macrophages while promoting myelin phagocytosis by microglia. On the other hand, IL-4 and IL-10 promote the phagocytosis of myelin by both microglia and macrophages [80]. The finding that microglia are more likely to phagocytose myelin at the single-cell level [28,36] is consistent with increased expression of IFN-γ, TNF-α, and IL-4, which promote phagocytosis by microglia. These cytokines may affect myelin phagocytosis by altering receptor expression, metabolism, and cytoskeleton dynamics in microglia and macrophages. However, conflicting results have also been reported on whether these cytokines enhance or inhibit the phagocytosis by microglia and macrophages [78].

It is possible that microglia and blood-derived monocytes affect each other’s phagocytic capacity. Greenhalgh et al. subjected CCR2-knockout (KO) mice to spinal cord injury and verified the phenotype of microglia in the absence of monocyte recruitment to the injury site [81]. Then, expression levels of pro-inflammatory cytokines and myelin phagocytosis by microglia were increased compared to those in wild type mice. Furthermore, using co-cultures of microglia and macrophages, the authors showed that the presence of macrophages suppressed myelin uptake by microglia, while the presence of microglia enhanced myelin uptake by blood-derived monocytes. These findings using cultured cells are consistent with the findings that macrophages phagocytose more myelin than microglia in vivo.

Other environmental factors that may contribute to differences in myelin phagocytosis between microglia and macrophages include the frequency and length of exposure to myelin phagocytosis. For example, it has been reported that microglia phagocytose myelin debris in the cortex, where constant myelin degeneration and remyelination occur under physiological conditions and with aging [60,61]. Thus, in the onset of pathogenesis, it is possible that microglia are already prepared for myelin phagocytosis and may have a higher phagocytic capacity than macrophages that first encounter myelin debris.

**Figure 2:**
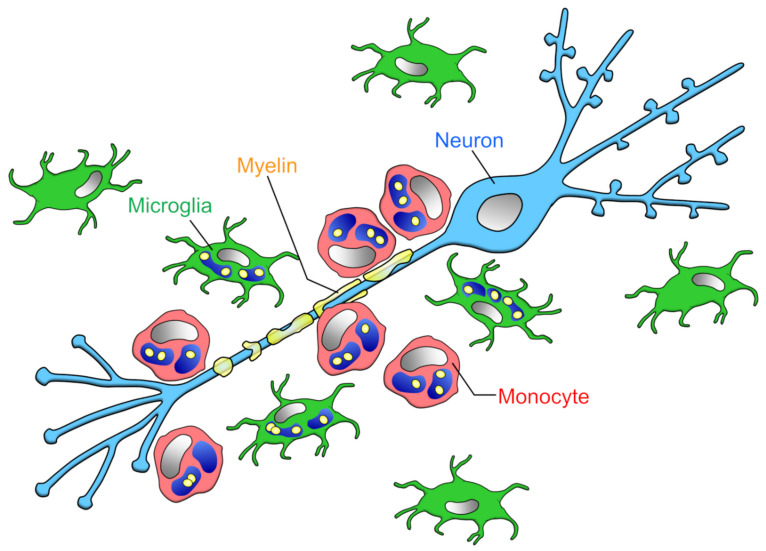
Phagocytic capacities of microglia and infiltrating monocytes in the early phase of MS: Yamasaki et al. reported that at the onset of EAE, the ratio of microglia to monocytes contacting demyelinated axons is about 1:2, monocytes locating closer to axons than microglia [15]. The amount of myelin phagocytosis per cell is higher in microglia than monocytes [76,77], but monocytes as a whole (i.e., per animal) phagocytose more myelin.

Finally, the comparison of myelin phagocytosis by microglia and blood-derived macrophages requires careful selection of validation methods. For example, flow cytometry of spinal cord samples showed that microglia and monocytes were equal in number during EAE onset, whereas the ratio of microglia to monocytes was 2:1 at the EAE peak [15]. However, as mentioned earlier, monocytes phagocytose more myelin than microglia in the vicinity of spinal cord axons, and it is not possible to determine which cell’s phagocytosis is dominant by simply comparing cell numbers (Figure 2).

### 3.2. Apoptotic Cells

Stroke is often accompanied by neuronal cell death, and the infiltration of blood-derived monocytes into the brain parenchyma has been identified. Dead cells and their debris that accumulate in the brain parenchyma elicit an excessive inflammatory response [82]. Therefore, early clearance of dead cells is essential for the restoration of brain homeostasis.

Ritzel et al. compared the properties of microglia and blood-derived monocytes (CD11b^+^, CD45^+^, LyC6 high expression (LyC6^high^)) after ischemic stroke caused by middle cerebral artery occlusion (MCAO) by using flow cytometry, which can separate the two cell types on the basis of their CD45 expression level [40]. The number of microglia in the brain parenchyma decreased from immediately after to 72 h after MCAO, while the number of monocytes increased. Consistent with this result, approximately 90% of the cells that were positive for BrdU, a proliferation marker, were monocytes. Furthermore, when the phagocytosis of microglia and macrophages isolated 72 h after MCAO was verified by the bead uptake assay, both the percentage of cells incorporating beads and the number of beads taken up by single cells were higher in monocytes. Although the phagocytosis of dead cells was not verified, it is possible that the phagocytosis by monocytes is dominant after stroke.

The blood-derived monocytes that invade the brain parenchyma after stroke are either inflammatory (CCR2^+^Ly6C^hi^) or responsible for tissue repair (CX3CR1^+^Ly6C low expression (Ly6C^lo^)). Garcia-Bonilla et al. examined the properties of infiltrating macrophages from 1 to 28 days after MCAO by flow cytometry [43]. Three days after MCAO, there were more CCR2^+^Ly6C^hi^ and no CX3CR1^+^Ly6C^lo^ cells, whereas the latter started to appear 14 days after MCAO. While CCR2-KO mice, in which the infiltration of CCR2^+^Ly6C^hi^ cells was suppressed, showed no accumulation of CX3CR1^+^Ly6C^lo^ cells, the populations of CX3CR1^+^Ly6C^lo^ cells increased with time after MCAO in *Nr4a1*-KO mice, which lacked CX3CR1^+^Ly6C^lo^ cells. These results suggest that CCR2^+^Ly6C^hi^ cells infiltrate in the early stage after MCAO transforms into CX3CR1^+^Ly6C^lo^ cells. Together with the above findings by Ritzel et al., it would be interesting if blood-derived monocytes with high phagocytic activity infiltrate during the period when dead cells are abundant and change their properties to tissue repair when the clearance of dead cells is completed.

**Figure 3:**
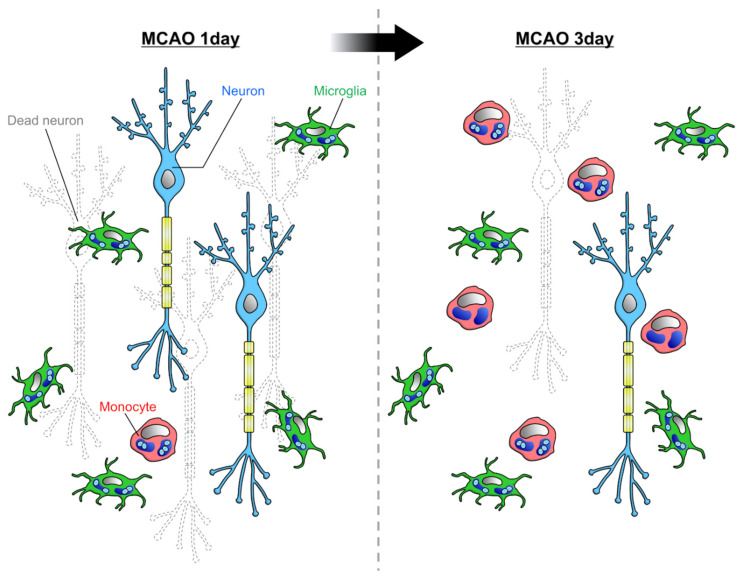
Phagocytic capacities of microglia and infiltrating monocytes in ischemia: Rajan et al. reported that at 1 day after MCAO, when neuronal cell death is active, the majority of phagocytes in the parenchyma are microglia [42]. As more time passes, the infiltration of monocytes increases, and the ratio of microglia to monocyte becomes about 5:4.

However, it has been suggested that microglia are more involved in the phagocytosis of dead cells in vivo [16]. Schilling et al. used bone marrow chimera mice to compare the phagocytic ability of microglia and blood-derived monocytes after MCAO. Microglial density increased from immediately after MCAO to 14 days later, while macrophage infiltration increased from 2 days after MCAO and decreased after 7 days. At 7 days after MCAO, when infiltration was most accelerated, the density of microglia was approximately three times higher than that of macrophages. The density of microglia that took up neurons was highest 1 day after MCAO and then decreased. Macrophages ingesting neurons were found 4 to 10 days after MCAO, but the phagocytosis of dead cells was mainly performed by microglia. This finding is consistent with previous studies showing that the order of phagocyte accumulation in the infarcted area after MCAO is microglia followed by blood-derived monocytes. Therefore, it is possible that microglia and blood-derived monocytes are responsible for the removal of dead cells and tissue repair, respectively. The reason why blood-derived monocytes seem to be less likely to phagocytose dead cells may simply be that the timing of infiltration into the brain parenchyma is when the removal of dead cells is almost complete. Another study examined the number of microglia and blood-derived monocytes by flow cytometry in a rat MCAO model [42]. The results showed that after 1 day of MCAO, the ratio of the number of microglia to monocytes was 8:1, whereas after 3 days of MCAO, the number of monocytes increased, and the ratio of the number of microglia to monocytes was 5:4 [42]. Together, it is likely that microglia are more numerous than monocytes at the time of active dead cell removal immediately after MCAO (Figure 3). However, Ritzel et al. reported that the ratio of the number of microglia to monocytes was 1:3 after 3 days of MCAO [40]. This difference might be caused by the difference in the severity of MCAO or the threshold of markers in flow cytometry.

### 3.3. Tumor Cells

Brain tumors are a typical environment where microglia and peripheral macrophages coexist. Tumor-associated macrophages and microglia, which are sometimes referred to as TAMs, are the major cell types that form tumors and have been shown to promote tumor growth. Therefore, it has been considered that controlling TAM function could suppress tumor growth. Actually, it was shown that suppressing SIRPα-CD47 signaling, which is a don’t eat me signal, and promoting tumor phagocytosis by TAMs could be a therapeutic approach for various tumors [83]. However, this antitumor effect has been thought to be mostly due to blood-derived monocytes infiltrating from the periphery, and the contribution of resident microglia has not been clarified. Hutter et al. attempted to elucidate this point by transplanting glioblastomas into CCR2^RFP/wt^CX3CR1^GFP/wt^ mice, in which microglia and macrophages can be distinguished by GFP or RFP expression [29]. Through the use of flow cytometry, these authors showed that the majority of cell types comprising TAMs were microglia. Next, they enhanced tumor phagocytosis by TAMs by treatment with anti-CD47 antibodies, which increased the percentage of microglia and macrophages that infiltrated and phagocytosed tumors. Furthermore, anti-CD47 antibody treatment increased microglial infiltration and tumor phagocytosis even in CCR^RFP/RFP^CX3CR1^GFP/wt^ mice in which macrophage infiltration into tumors was absent. The survival of CCR2^RFP/RFP^CX3CR1^GFP/wt^ mice was significantly prolonged after treatment with anti-CD47 antibody, suggesting that tumor phagocytosis by microglia contributes to recovery from brain tumors. By comparing these results with the tumor phagocytosis and survival of macrophages in mice in which microglia were specifically removed from the brain parenchyma, it will be possible to verify whether microglia or macrophages are more likely to phagocytose tumors.

However, Chen et al. reported that microglia accounted for approximately 12% and monocytes and macrophages together accounted for approximately 83% of cells in the TAMs of a mouse glioblastoma model [41]. Thus, although the term “glioblastoma” is widely used, the proportion of cell types in TAMs differs among the papers. This difference may be due to various factors, such as the model used and the degree of tumor progression at the time of analysis, and detailed validation will be required to determine whether microglia, monocytes, or macrophages predominate in tumor phagocytosis.

In Section 3.1, Section 3.2 and Section 3.3, we discussed the comparison of the phagocytic capacity of microglia and blood-derived monocytes in diseases in which monocyte infiltration into the brain parenchyma has been confirmed. In addition to these acute CNS diseases, it has been suggested that blood-derived monocytes infiltrate the brain parenchyma in chronic diseases such as Alzheimer’s disease (AD) and viral infections. However, due to the difficulty in distinguishing between microglia and blood-derived monocytes, there is debate as to whether or not blood-derived monocytes infiltrate the parenchyma. In the next section, we introduce studies that examined phagocytosis by microglia and blood-derived monocytes in AD or virus-infected brains.

### 3.4. Amyloid-β (Aβ)

In the cortex and hippocampus of AD brains, Aβ deposition leads to the formation of Aβ plaques. These Aβ plaques are a pathological hallmark of AD, and aggregated Aβ exerts cytotoxic effects, including ion channel blockage, calcium homeostasis disruption, mitochondrial oxidative stress, and energy metabolism impairment [84,85]. Since microglia are the major phagocytes in the brain parenchyma, the phagocytosis of Aβ by microglia has been considered important for the clearance of Aβ and thus for the treatment of AD [86]. However, there is some controversy as to whether microglia phagocytose Aβ. For example, in vitro experiments have shown that microglia uptake Aβ [87,88], but microglia may not have the ability to digest Aβ [89]. In vivo, although many studies have observed microglial uptake of Aβ [88,90], it has been also indicated that microglia are not involved in Aβ clearance because microglial depletion affected neither the number nor size of Aβ plaque [23]. In addition to microglia, perivascular and meningeal macrophages have been suggested to suppress Aβ deposition in the brain parenchyma by removing Aβ from the blood and spinal fluid through phagocytosis [25,26]. It should be noted, however, that the authors of the original articles argued that it is not possible to determine which state of Aβ is being phagocytosed by microglia and monocytes: monomer, oligomer, fibril, or plaque. This is because Aβ phagocytosis by microglia and monocytes has been verified by changes in the number and size of Aβ plaques.

Several studies have reported the contribution of circulating monocytes and infiltrating macrophages to Aβ removal. Hawkes et al. took advantage of the fact that clodronate administration to the lateral ventricles specifically eliminates perivascular macrophages (defined by CD206 and CD163 expression) to examine the effect of macrophages on Aβ in the leptomeninges and blood vessels [25]. The administration of clodronate to TgCRND8 mice, one of the mouse models of AD, significantly increased the amount of Aβ in cortical blood vessels. In addition, when macrophage turnover was promoted by chitin, a component of the cytoskeleton of crustaceans and insects, Aβ in cortical blood vessels was significantly reduced. Fiala et al. compared the amount of Aβ phagocytosis by monocytes and macrophages from the blood of healthy subjects and patients with AD and found that cells from AD patients had a lower phagocytic capacity [91]. These results indicate that macrophages are able to phagocytose Aβ and that impaired phagocytosis of Aβ by macrophages may contribute to the development of AD.

Does the phagocytosis of Aβ in blood vessels by monocytes affect the deposition of Aβ in the brain parenchyma? Michaud et al. observed the behavior of circulating monocytes toward Aβ in blood vessels by in vivo two-photon live imaging [26]. The authors used mice expressing GFP under the CX3CR1 promoter, a fractalkine receptor expressed on immune cells, including monocytes and microglia, and detected monocytes circulating in blood vessels by GFP. Interestingly, among arteries, veins, and veins with Aβ, circulating monocytes were more likely to stay in veins with Aβ. The authors also found that circulating monocytes took up Aβ in the blood vessels. In addition, the contribution of circulating monocytes (defined by low expression of Ly6C) was examined through the creation of blood chimeras between APP/PS1 mice, a mouse model of AD, in which bone marrow was removed by the administration of the antineoplastic drug busulfan/cyclophosphamide, and *Nr4a1*-KO mice, in which circulating monocytes were absent. The results showed that the area of Aβ plaques in the cortex and hippocampus was increased in the group of mice with APP/PS1-*Nr4a1*-KO blood chimeras compared to the area in the group of mice with APP/PS1-wild-type blood chimeras after bone marrow removal. These results suggest that monocytes inhibit Aβ deposition in the brain parenchyma by phagocytosing Aβ in blood vessels.

The role of microglia and blood-derived monocytes in the clearance of Aβ in the hippocampus was examined [24]. When bone marrow cells from CAG-EGFP mice, in which almost all the cells express EGFP, were intravenously administered to APP/PS1 mice that had been irradiated to remove peripheral macrophages, GFP^+^ cells infiltrated the hippocampus, accumulated around Aβ plaques, and phagocytosed Aβ. Furthermore, to directly examine the role of blood-derived monocytes, APP/PS1 mice, a typical mouse model of AD, were crossed with CD11b-HSVTK mice, which express thymidine kinase under the CD11b promoter and can eliminate CD11b^+^ phagocytes in a ganciclovir-dependent manner. Ganciclovir was administered into the ventricles of the crossed mice to suppress the infiltration of myeloid cells into the brain parenchyma. This resulted in an increase in the number of Aβ plaques in the hippocampus, despite the microglial accumulation around Aβ plaques. These results suggest that blood-derived monocytes are more capable of phagocytosing Aβ than microglia. In their previous report, the authors claimed that blood-derived monocytes differentiate into microglia because the GFP^+^ cells infiltrating into the brain parenchyma by this method were Iba1^+^ and exhibited a ramified morphology [24]. However, it is not accurate to conclude that GFP^+^ cells are microglia based on these characteristics alone, and it should be noted that these authors have not verified the expression of potential microglia-specific genes such as *P2Y12* and *TMEM119*. Furthermore, since irradiation itself promotes monocyte infiltration into the parenchyma [92,93], it is possible that Aβ phagocytosis by blood-derived monocytes is overestimated.

In light of the above papers, it is expected that blood-derived monocytes are more capable of phagocytosing Aβ than microglia. Grathwohl et al. crossed APP/PS1 mice with CD11b-HSVTK mice and achieved microglia-specific removal by adjusting the concentration of ganciclovir administered into the ventricle [23]. Ganciclovir administration did not change the number or size of Aβ plaques in the cortex or hippocampus, suggesting that microglia are not capable of clearing Aβ. The authors claimed that there was no macrophage infiltration at the time and in the brain regions observed in this paper, and they did not examine the contribution of blood-derived monocytes to the clearance of Aβ. It should also be noted that the study did not examine whether ganciclovir administration affects the survival and proliferation of blood-derived monocytes.

Microglia have been considered to exert neuroprotective function because they inhibit Aβ expansion by surrounding and phagocytosing Aβ plaques [94]. Recently, however, it has been suggested that the phagocytosis of Aβ by microglia even promotes the formation of Aβ plaques [90], and the brain may prevent the formation of Aβ plaques by delegating the phagocytosis of Aβ to blood-derived monocytes. What is the molecular basis of the difference in Aβ phagocytosis between microglia and blood-derived monocytes? Transforming growth factor β (TGF-β)-mothers against decapentaplegic homolog (Smad) 2/3 signaling has been reported to have opposing effects on microglia and blood-derived monocytes in Aβ phagocytosis. Tichauer et al. showed that TGF-β1-Smad3 signaling enhances Aβ phagocytosis by microglia using isolated microglial cultures [95]. To examine the effect of TGF-β signaling in blood-derived monocytes on AD pathology, Town et al. crossed mice expressing a dominant-negative form of TGF-β receptor II in immune cells under the control of the CD11c promoter with Tg2576 mice [32]. The suppression of TGF-β signaling in blood-derived monocytes (CD11b^+^, CD11c^+^, CD45^+^, LyC6^low^) reduced Aβ deposition in the brain parenchyma and cerebral blood vessels by approximately 90%. The KO of TGF-β receptor II also increased the number of blood-derived monocytes that took up Aβ in the brain parenchyma. Similarly, in cultured monocytes, the KO of TGF-β receptor II enhanced Aβ phagocytosis. Furthermore, the KO of TGF-β receptor II promoted the activation of Smad1/5/8, suggesting that Smad1/5/8 is important in promoting Aβ phagocytosis by blood-derived monocytes. A similar trend was observed in the inhibition of TGF-β receptor I. However, since CD11c expression is upregulated in microglia surrounding Aβ plaques [31], it should be noted that it is not possible to accurately distinguish microglia from blood-derived monocytes depending on the expression of these markers.

Among the receptors involved in phagocytosis of Aβ, scavenger receptor class A (SR-A) may explain the difference between microglia and blood-derived monocytes. For example, Smad3 increases the expression of SR-A in microglia [95], and Smad2 signaling decreases the expression of SR-A in macrophages (derived from human monocytes, no molecular marker information). Smad3 is downregulated in Alzheimer’s patients and aging mice [95,96,97]; it makes sense that blood-derived monocytes are more capable of phagocytosing Aβ than microglia in the AD brain. However, the relationship between Smad2/3 and the expression of SR-A should not be simplified, because there are many conflicting results about the Smad2/3 activity in aging and AD [98].

**Figure 4:**
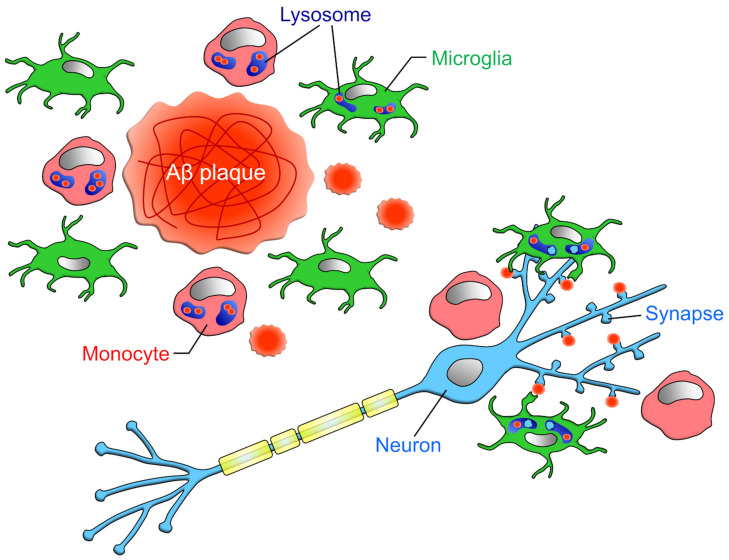
Phagocytic capacities of microglia and infiltrating monocytes in AD: Unger et al. has reported that in the vicinity of Aβ plaque, the ratio of microglia to monocytes is about 1:1 [17]. However, when restricted to CD68-positive cells, the ratio of microglia to monocyte was about 1:3. Microglia phagocytose not only Aβ but also synapses attached to Aβ, while monocytes do not phagocytose synapses [34].

Another important factor in determining whether microglia or monocytes are more capable of phagocytosing and removing more Aβ from the brain parenchyma is how many monocytes infiltrate the brain. However, few papers have compared the numbers of microglia and monocytes in the brain parenchyma. This is because the methods used to distinguish between microglia and monocytes, such as microglial removal and post-irradiation bone marrow transplantation, promote the infiltration of peripheral cells into the brain parenchyma [92,93]. Unger et al. attempted to distinguish microglia and monocytes by the expression level of CD45 and TMEM119 in APP/PS1 mice [17]. In the whole brain, the ratio of microglia to monocytes was 9:1, but in the hippocampus and cortex, where Aβ aggregation was observed, the ratio of microglia to monocytes was almost equal. Furthermore, the number of cells expressing CD68 (a lysosomal marker), which is an indicator for phagocytosis prediction, was 1:3 for microglia and monocytes in the hippocampus, implying that monocytes may be more capable of phagocytosing Aβ (Figure 4). However, this finding needs to be verified in various other animal models of AD. In addition, since CD45 expression is upregulated [37] and TMEM119 expression is downregulated in microglia under the inflammatory condition [35], it should be noted that whether the expression levels of these markers accurately distinguish microglia and blood-derived monocytes needs to be interpreted carefully.

As will be discussed in the following chapter on synapses, it has been shown that in AD brains, microglia phagocytose synapses in addition to Aβ, which may accelerate the deterioration of the pathology. On the other hand, the enhancement of Aβ phagocytosis by monocytes has not been shown to cause synapse loss, suggesting that the promotion of monocyte infiltration may be an effective treatment for AD.

### 3.5. Synapses

Microglia have been reported to phagocytose surplus synapses in the developing lateral geniculate nucleus and hippocampus, thereby contributing to the refinement of neural circuits and normal brain function [72]. In recent years, it has been confirmed that microglia phagocytose synapses in brains affected by neurodegenerative diseases such as AD and MS [72]. On the other hand, the infiltration of blood-derived monocytes into the brain parenchyma has been observed in these diseases, and it is possible that blood-derived monocytes also phagocytose synapses. Therefore, in this chapter, we will discuss the different roles of microglia and blood-derived monocytes in synaptic phagocytosis.

Microglial phagocytosis of synapses in the brains of AD model mice was first reported by Hong and colleagues [34]. The authors showed that synaptic phagocytosis by microglia is facilitated by the deposition of C1q at synapses in the J20 mouse model of AD and in mice treated with Aβ oligomers. Because Aβ binds to synaptic proteins [99,100], it is possible that Aβ anchors synapses and C1q to facilitate synaptic phagocytosis by microglia. It was not made clear whether blood-derived monocytes infiltrate into the brain parenchyma, but the authors confirmed that the cells expressing the complement receptors for synaptic phagocytosis also expressed P2Y12, a potential microglia-specific marker, suggesting that complement-dependent synaptic phagocytosis was mainly carried out by microglia (Figure 4).

Several studies have shown that blood-derived monocytes that infiltrate the brain parenchyma phagocytose Aβ in AD, but their involvement in synaptic phagocytosis is unclear. Using primary cultures of cortical neurons, Li and colleagues showed that Aβ fibrils and oligomers cause a loss of excitatory synapses and abnormalities in neurite morphology [38]. They found that these neuronal abnormalities caused by Aβ can be rescued by co-culturing neurons with bone-marrow-derived monocytes and bone-marrow-derived macrophages (bone marrow cells differentiated by RPMI-1640, FBS, and M-CSF treatment). They also showed that macrophages clear Aβ by phagocytosis and extracellular secretion of enzymes and that the activation of macrophages by glatiramer acetate (GA) promotes this clearance. Furthermore, a transplantation of blood-borne bone-marrow-derived monocytes (CD115^+^ and CD45^high^) into APP/PS1 mice rescued the reduction in the number of excitatory synapses in the olfactory cortex and hippocampus, indicating that infiltrative macrophages have a neuroprotective function in vivo. Consistent with the authors’ previous report that GA treatment promotes macrophage infiltration [101], GA treatment in a mouse model of AD showed neuroprotective effects as well as monocyte transplantation. Together with the results of in vitro and in vivo experiments, the authors consider that the neuroprotective effect of blood-derived monocytes is due to both the promotion of synaptogenesis and the inhibition of synapse loss through the clearance of Aβ. Furthermore, the correlation between the degree of infiltration of blood-derived monocytes and cognitive performance in a mouse model of AD supports the neuroprotective effects of blood-derived monocytes. Indeed, osteopontin, whose expression level is increased in monocytes and macrophages by GA treatment, has been shown to promote synaptogenesis [102,103]. These results suggest that blood-derived monocytes phagocytose Aβ but not synapses and that the phagocytosis of blood-derived monocytes may be an effective therapeutic target in AD. However, it should be noted that this study did not confirm the infiltration of CD115^+^ monocytes into the parenchyma; Wang et al. concluded that peripheral macrophages do not accumulate around Aβ plaques after parabiosis [104]. Furthermore, Di Liberto et al. showed that blood-derived monocytes can phagocytose synapses [18]. Thus, it is also possible that blood-derived monocytes phagocytose synapses in AD brain.

Viral infection causes an infiltration of circulating monocytes into the brain parenchyma. In a mouse model of West Nile virus (WNV) infection, Vasek et al. tested hippocampal-dependent cognitive decline and the possibility of synapse loss in this model [39]. In the hippocampus of WNV-infected mice, macrophage responses, such as increased Iba1 area and CD68 expression, were observed. In addition, WNV infection increased the expression of complement-pathway-related genes (*C1qa* and *C3*) in the hippocampus and decreased synaptic density in the CA3 field due to increased presynaptic-specific phagocytosis by microglia. Furthermore, viral infection increased the colocalization of presynapses with C1qA, and the decrease in synaptic density was blocked by the KO of *C3* and *C3aR* (the receptor of C3a, the cleavage product of C3), indicating the involvement of complement and subsequent synaptic phagocytosis by microglia. Importantly, flow cytometry revealed that the number of macrophages (CX3CR1^+^, CD45^high^, and CD11b^high^) infiltrating the brain parenchyma decreased to almost zero between 7 and 25 days after WNV infection, when synaptic phagocytosis and synaptic density reduction were observed. Therefore, the WNV-induced synaptic phagocytosis observed in the study was likely to be mainly performed by microglia.

Synaptic phagocytosis has also been observed after *Toxoplasma* infections. Carrillo et al. showed that hippocampal microglia and infiltrating monocytes in a mouse model of *Toxoplasma* infection stripped and phagocytosed inhibitory presynaptic terminals around the cell body [44]. Using CX3CR1-GFP mice, in which both microglia and circulating monocytes express GFP, the authors found that there was a mixture of cells with high and low GFP fluorescence intensity in the brain parenchyma. Interestingly, the cells that surrounded the neuronal cell bodies had high GFP fluorescence. As Yamasaki et al. suggested that cells with high GFP fluorescence intensity were resident microglia and those with low fluorescence intensity were infiltrating monocytes (F4/80^+^, CCR2^+^) [15], it is possible that microglia predominantly phagocytosed synapses after *Toxoplasma* infection. Notably, microglia in the infected mice had enlarged cell bodies with thin processes, but the infiltrating monocyte-like cells had few processes, suggesting the possibility that microglia had higher surveilling ability. Since previous studies have reported that *Toxoplasma* infection activates the complement pathway [105,106], it is possible that *Toxoplasma*-induced synaptic phagocytosis is also complement-dependent.

It has also been reported that infiltrating monocytes may phagocytose synapses. Di Liberto et al. used a mouse déjà vu model to examine the interaction between CD8^+^ T cells and phagocytes in synapse loss during viral infection [18]. The déjà vu model is an animal model that exhibits pathological features (microglial activation and infiltration of CD8^+^ T cells) similar to Rasmussen’s encephalitis, a rare central inflammatory disease. Experimentally, the inflammatory response is elicited by administering weakened lymphocytic choriomeningitis virus to the cortex of neonatal mice and again intravenously in adolescence. In the infected mice, the numbers of inhibitory pre- and postsynaptic terminals formed in the deep cerebellar nuclei neurons were reduced. Around the cell body of virus-infected neurons, activated Iba1^+^ phagocytes (with enlarged and shortened processes) took up presynaptic proteins. Interestingly, some of the Iba1^+^ cells did not express TMEM119, a potential microglia-specific marker, suggesting that blood-derived monocytes may also phagocytose synapses. However, because TMEM119 expression was reported to be reduced in microglia of diseased brains [35], it is difficult to distinguish between microglia and monocytes based solely on the TMEM119 expression.

A similar experimental paradigm was performed using CX3CR1^CreERT2/+^ x R26-Stop-RFP^fl/+^ mice, in which microglia and monocyte-derived cells could be distinguished by the presence or absence of RFP expression [45]. This approach takes advantage of the fact that temporary tamoxifen administration causes both microglia and monocytes to be labeled with RFP, but the turnover of monocytes is faster than that of microglia, allowing RFP to be identified only in microglia. The authors found that RFP-negative monocytes also surrounded the cell bodies of infected neurons. Furthermore, infection-induced synaptic phagocytosis was not rescued in C3/C4-KO mice, suggesting that synaptic phagocytosis in this mouse model is independent of the complement pathway. The authors also demonstrated a molecular mechanism by which IFN-γ released from CD8^+^ T cells increases CCL2 expression in neurons via STAT1, and phagocytes that receive CCL2 engulf synapses. Although they did not compare the amount of synaptic phagocytosis between microglia and blood-derived monocytes, since CCR2 is mainly expressed in blood-derived monocytes [107], synaptic phagocytosis in the déjà vu model may be dominated by monocytes. In fact, in this study, the depletion of monocytes through the administration of MC-21, an anti-CCR2 antibody, restored motor function and synapse loss caused by viral infection, suggesting that synaptic phagocytosis by monocytes contributes significantly to pathology.

Thus, the complement cascade would rather affect microglial engulfment of synapses than that of blood-derived monocytes. Among the complement receptors, CR3 is the one most associated with synaptic phagocytosis. As it is expressed in both microglia and macrophages, the difference in complement-dependent synaptic phagocytosis between the two may be due to the localization (e.g., more at the tip of the projection in microglia) rather than the amount of CR3 expression. As mentioned above, in the study by Carrillo et al. suggesting complement-dependent synaptic phagocytosis by *Toxoplasma* infection, the infiltrating monocyte-like cells had few ramified processes and did not phagocytose synapses, while the study by Di Liberto et al. suggested complement-independent synaptic phagocytosis in the déjà vu model; infiltrating monocytes had short processes and phagocytosed synapses [18,44]. It is still speculative, but it is possible that complement-dependent synaptic phagocytosis requires finely branched processes and the localization of complement receptors there.

Few studies have verified the number of microglia and monocytes in the brain parenchyma of animal models of viral infection. Vasek et al. measured the number of these cells by flow cytometry of whole-brain samples of mice infected with WNV [39]. The numbers of microglia were approximately 200 × 10^2^ (control), 160 × 10^2^ (8 days after infection), and 160 × 10^2^ (25 days after infection), while the numbers of monocytes were 0 (control), 50 × 10^2^ (8 days after infection), and 5 × 10^2^ (25 days after infection). For viral infection models, it should be noted that the degree of BBB disruption and the type and amount of proinflammatory cytokines secreted by each model may differ, resulting in different degrees of monocyte infiltration, which may determine whether monocytes can phagocytose synapses.

## 4. Significance of Phagocytosis on Phagocytes

**Figure 5:**
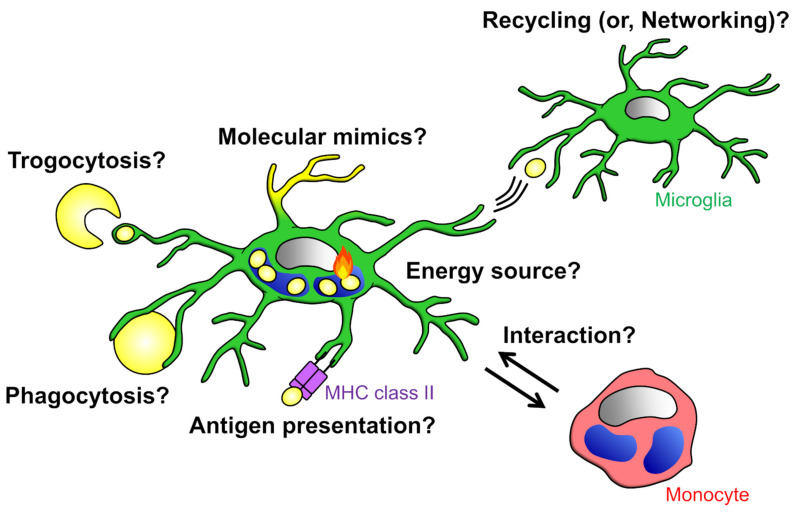
Significance of phagocytosis: Phagocytes possibly depend on the energy produced by the degradation of phagocytosed materials for their survival. It is also possible that phagocytes expose phagocytosed materials to the cell surface to mimic the target of phagocytosis. It has been suggested that phagocytes network or interact with other cells via presentation and exchange of phagocytosed materials.

The mechanism by which microglia and blood-derived monocytes remove neurotoxic substances has been the subject of accumulating research, with the expectation that it will lead to the prevention and amelioration of brain diseases. Differences in phagocytic mechanisms induced by different phagocytic targets and phagocytosis amounts can be reflected in intracellular signaling within phagocytes, and thus may affect phagocyte functions. Therefore, in Chapter 4, we will generalize a little and review the significance of phagocytosis for phagocytes, to help understand the difference between microglia and blood-derived monocytes (Figure 5). It possibly contributes to the manipulation of phagocyte functions through the regulation of phagocytosis, and eventually to the discovery of therapeutic targets. Further, in this chapter, we will discuss the possibility that phagocytosis has significance beyond the mere removal of unwanted materials from the CNS.

### 4.1. Suppression of Inflammatory Response

Several studies have reported that inflammatory responses of macrophages are suppressed after phagocytosis [108]. Most of these reports evaluated the inflammatory response by treating LPS to macrophages co-cultured with myelin debris or apoptotic cells and comparing the release of various cytokines. With these validation methods, however, it is not possible to separate the recognition, uptake, and degradation of phagocytic targets. Indeed, Kim and Ma showed that contact with apoptotic cells alone inhibited or promoted the release of the pro-inflammatory cytokine IL-12 or the anti-inflammatory cytokine TGF-β by macrophages, respectively [109]. Different approaches are needed to examine the impact of intracellular degradation products resulting from phagocytosis on phagocyte function, and such studies are presented below.

Bogie et al. showed that phosphatidylserine (PS), a phospholipid contained in myelin, causes LXRs-dependent suppression of the release of pro-inflammatory cytokine IL-6 after myelin phagocytosis by macrophages in vitro [110]. In a following report by the same group, it was shown that myelin-derived PS causes PPARs-dependent suppression of NO release from phagocytosing macrophages [111]. The authors further showed that intravenous administration of PS-containing liposome to EAE model rats induced uptake of PS by macrophages in the demyelinated area and alleviated the symptoms of EAE. Furthermore, the authors examined in more detail the changes in macrophage properties after myelin phagocytosis [112]. By analyzing cultured cells and MS patient brains, they found that myelin uptake inhibited the release of pro-inflammatory cytokines, but it was promoted with increasing myelin exposure time. SCD1, an enzyme that converts saturated fatty acids to monounsaturated fatty acids, was suggested to be involved in the switch from anti-inflammatory to inflammatory phenotype. In addition, monounsaturated fatty acids were found to promote the inflammatory response by inhibiting ABCA1-dependent extracellular release of cholesterol.

It has been suggested that suppression of the inflammatory response of macrophages is also caused by phagocytosis of apoptotic cells. Morioka et al. found that gene expression levels of several solute carrier (SLCs) were increased and the glycolytic system was promoted during the process of phagocytosis by macrophages [113]. It was also suggested that among SLCs, SLC16A1, whose expression increases upon uptake of apoptotic cells, promotes the release of lactate, a product of the glycolytic system, and changes the surrounding macrophages into anti-inflammatory state. Zhang et al. found that fatty acids derived from apoptotic cells altered the properties of macrophages [114]. Fatty acids in macrophages, which increase with apoptotic cell phagocytosis, promoted mitochondrial respiration and NAD^+^-dependent signaling cascades. This in turn contributes to cardiac tissue repair by promoting the production and release of the anti-inflammatory cytokine IL-10 through the activation of SIRT1 and binding of the transcription factor Pbx1 to the IL-10 promoter.

### 4.2. Acquisition of Nutrients

Once transported to the digestive organelle lysosome, phagocytosed materials are degraded by various enzymes [115]. This degradation reaction is accompanied by a heat release reaction, which indicates that energy is produced in the cell after phagocytosis. It is possible that phagocytes depend on the energy produced by the degradation reaction for their survival. In particular, these cells move their cytoskeletons dynamically, for example, during migration and in the formation of phagocytic cups [116], and they are expected to consume a large amount of energy compared to nonphagocytic cells. Phagocytes may phagocytose to obtain energy for further phagocytosis. Findings by Yurdagul et al. would support this idea: They found that digestion of phagocytosed materials is important for sustained phagocytosis by macrophages [117]. Macrophages metabolize dead cell-derived arginine into ornithine and putrescine, and putrescine increases the expression of Dbl (Rac1 guanosine triphosphatase (GTP)-exchange factor). Dbl activates Rac1 and promotes actin-dependent uptake of apoptotic cells. It has also been suggested that myelin phagocytosis increases the expression level of CD36 in microglia and macrophages, promoting further myelin phagocytosis [118]. Microglia continuously extend and retract their processes to monitor the surrounding environment, and it would be interesting to investigate whether the energy obtained from phagocytosis is also utilized in this constant surveillance.

### 4.3. Survival in Unfamiliar Environments

Miller et al. previously showed that *Entamoeba histolytica* invades the human body and trogocytoses human cells [119]. In this paper, the authors found that *Entamoeba histolytica* exposes fragments of human cells to the cell surface, thereby escaping lysis by human serum [120]. Does this defense mechanism of coating oneself with phagocytosed materials to survive in an environment composed of cells of different origins also work in phagocytes? For example, microglia infiltrate into the brain parenchyma from the yolk sac during embryonic development, and it is possible that microglia, which are originally derived from the mesoderm, have some kind of defense or mimicry mechanism to survive in the ectoderm-derived brain environment. It has been shown that microglia phagocytose dead neurons during invasion and that the inhibition of neuronal cell death suppresses microglial invasion into the brain parenchyma [121,122]. Taken together, these results and those reported by Miller et al. raise the possibility that microglia can survive in an ectoderm-derived environment by exposing phagocytosed dead cell debris to the cell surface.

### 4.4. Antigen Presentation

It has been shown that phagocytosis can be used as a tool for intercellular communication, and a representative example is antigen presentation. Dendritic cells are the most common type of cells that present antigens, but a number of papers have argued that microglia and macrophages also have the potential to present antigens [123]. However, it should be noted that most of the findings from these studies are based solely on the presence or absence of expression of antigen presentation-related proteins such as MHC class II. In a recently published paper, Mundt et al. examined the ability of dendritic cells, B cells, microglia, and macrophages to present antigens using myelin debris [124]. The authors tested the possibility that each cell type presents antigens by knocking out antigen-presentation-related proteins specifically in these cell types, and they showed that dendritic cells, but not microglia or macrophages, regulate T cell activation via antigen presentation.

Thus, microglia and macrophages may not be the major antigen-presenting cells, but since microglia secrete extracellular vesicles, it is possible that they pass antigen-encapsulating vesicles between neighboring cells to transmit information.

### 4.5. Networking

It was also shown that phagocytosed materials can be exchanged not only between proximal cells but also between distal cells. For example, it has been suggested that microglia release phagocytosed tau and contribute to tau propagation [125]. This may function as an alert to inform phagocytes in far brain regions of abnormalities occurring locally. On the other hand, a study examining tau propagation by infiltrating macrophages in the brain parenchyma of mice in which microglia were removed showed no change in the degree of tau accumulation. Therefore, it was concluded that macrophages do not contribute to tau propagation [126]. Since some studies reported that peripheral macrophage-derived exosomes enter the brain parenchyma [127], it is likely that macrophages are capable of releasing phagocytosed material. Microglia are also known to exocytose lysosomes [128], and it is expected that the phagocytosed materials encapsulated in vesicles are exchanged. There are still many unanswered questions about the existence and mechanism of signal transduction by extracellularly released vesicles and whether it can occur between heterogeneous cells. In recent years, a variety of tools have been developed to examine contact- and noncontact-dependent cell–cell interactions [129,130,131]; therefore, it is expected that the study of cell–cell interactions via phagocytosis will be significantly developed.

Although many studies have shown that the metabolites of phagocytosis alter the inflammatory properties of phagocytes, few studies supported the relationship between phagocytosis and the survival, antigen presentation, and network formation of phagocytes, as described in the latter part of this chapter. Further studies on the effects of phagocytosis on phagocytes will not only elucidate the biological properties of phagocytes, but also contribute to develop the method to manipulate the function of phagocytes, and thus to the discovery of therapeutic targets.

## 5. Conclusions and Outlook

Many unanswered questions remain. One question of particular interest to us is why circulating monocytes need to be recruited to the parenchyma despite the large numbers of brain-resident macrophages, i.e., microglia. Infiltration of monocytes can be merely the result of a breakdown of BBB. Another possibility is that recruiting cells that have different phagocytic capacities (the expression of receptors required for the recognition of phagocytic targets, amount of phagocytosis, digestive capacity, etc.) from microglia to the parenchyma may be effective in removing unwanted materials. It may also be important that the infiltrating monocytes reside in areas that are inherently isolated from the parenchyma and are not exposed to cytokine storms caused by the damage or inflammation occurring in the parenchyma. Indeed, as discussed in the section on Aβ phagocytosis, ambient cytokine levels have been shown to affect the expression and function of phagocytic receptors. Furthermore, the fate of monocytes that infiltrate the brain parenchyma is still unknown. It is possible that after exerting their phagocytic activity in the brain parenchyma, the monocytes may re-enter the circulatory system, but given the short lifespan of circulating monocytes [19], they are more likely to die in the parenchyma. If this is the case, the impact of the processing mechanism of dead monocytes on pathogenesis will be an important issue. Additionally, as mentioned in the last chapter, clarifying the changes in the properties of phagocytes upon phagocytosis will help to predict the effects (both good and bad) of phagocytosis manipulation as described above.

Finally, in this review, the phagocytic functions of microglia and infiltrating monocytes in the CNS parenchyma were outlined for comparison. We also found that it is difficult to make a general comparison of the magnitude of the contribution of each phagocyte to the clearance of unwanted debris because the mechanism of phagocytosis is affected by multiple factors, including the type of disease, the target of phagocytosis, and the timing and ability of microglia and monocytes to exert their phagocytic abilities. In the future, it is hoped that the phagocytic capacity of microglia and circulating monocytes will be examined under the same conditions in the same animal and that the differences between them will be clarified. Recently, induced-pluripotent-stem-cell-derived microglia [132,133,134,135,136,137] and blood-derived microglia-like cells [138,139] were developed. If these technologies can be applied to manipulate the CNS environment through efficient enhancement or inhibition of phagocytosis, they will lead to the prevention and treatment of diseases such as those described in this review.

## Figures and Tables

**Table 1:** Transgenic animals for immune cell subtype-specific analysis.

Animal	Manipulation	Objective	Limitation	Reference
CD11b-HSVTK mice	Injection of GCV into the ventricle	Microglia-specific depletion	Long-term administration of GCV causes microhemorrhages and artificial influx of peripheral macrophages into the CNS [23].	[23]
		Inhibition of monocyte infiltration into the brain parenchyma		[24]
unspecified	Injection of clodronate into the lateral ventricle	Perivascular-macrophage-specific depletion	-	[25]
*Nr4a1^−/−^* mice	BM derived monocyte transplantation	BM derived monocyte-specific labeling	-	[26]
CX3CR1^CreERT2/+^xR26-stop-RFP^fl/+^ mice	Intraperitoneal injection of tamoxifen	Microglia-specific labeling	It is necessary to distinguish between microglia and BAMs [27].	[18]
β-actin-EGFP mice	BM derived macrophage transplantation	BM derived macrophage-specific labeling	-	[28]
CCR2-RFP::CX3CR1-GFP mice	-	Distinguishment between microglia and macrophage (microglia: GFP; monocyte: RFP)	-	[15,29]
lysozyme M EGFP-knockin mice	BM derived monocyte transplantation	BM derived monocyte-specific labeling	-	[30]
C57BL/6J-GFP mice	BM derived monocyte transplantation	BM derived monocyte-specific labeling	-	[16]
CD11c-DNR mice		BM derived monocyte-specific manipulation	CD11c expression is upregulated in microglia surrounding Aβ plaques [31].	[32]

**Table 2:** Marker molecules for immune cell subtype-specific analysis.

Marker	Observation System	Objective	Limitation	Reference
P2Y12	Immunohistochemistry	Microglia-specific detection	In the disease conditions, P2Y12 is downregulated in microglia [33].	[34]
TMEM119	Immunohistochemistry	Microglia-specific detection	In the disease conditions, TMEM119 is downregulated in microglia [35].	[17,18]
Il-69	Immunohistochemistry	Hematogenous macrophage-specific detection	-	[36]
CD45	Flow cytometry	Distinguishment between microglia and BM-derived monocyte (low: microglia; high: monocyte)	In the disease conditions, CD45 is upregulated in microglia [37].	[38,39,40,41,42]
CX3CR1	Flowcytometry	Distinguishment between microglia and BM-derived monocyte (low: monocyte; high: microglia)	In the disease conditions, CX3CR1 is downregulated in microglia [37].	[39,43]
	Immunohistochemistry			[44]
CCR2	Flow cytometry	Distinguishment between microglia and BM-derived monocyte (low: microglia; high: monocyte)	-	[43]
Ly6C	Flow cytometry	Distinguishment between microglia and BM-derived monocyte (low: microglia; high: monocyte)	Ly6C^hi^ monocytes give rise to Ly6C^lo^ monocytes [45,46].	[40,41,43]

## Data Availability

Not applicable.

## Abbreviations

Aβ	amyloid-β
ABCA1	ATP-binding cassette transporter A1
AD	Alzheimer’s disease
BAMs	border-associated macrophages
BBB	blood brain barrier
BM	bone marrow
BrdU	bromodeoxyuridine
C1q	complement component 1q
C3	complement component 3
C3aR	C3a anaphylatoxin receptor
C4	complement component 4
CAG	CMV early enhancer/chicken β actin
CCL2	CC chemokine ligand 2
CCR2	CC chemokine receptor 2
CD8	cluster of differentiation 8
CD11b	cluster of differentiation 11b
CD11c	cluster of differentiation 11c
CD11c-DNR	CD11c promoter–driven dominant-negative TGF-b receptor type II
CD36	cluster of differentiation 36
CD45	cluster of differentiation 45
CD47	cluster of differentiation 47
CD68	cluster of differentiation 68
CD93	cluster of differentiation 93
CD115	cluster of differentiation 115
CD163	cluster of differentiation 163
CD206	cluster of differentiation206
CNS	central nervous system
CR3	complement component 3 receptor
CSF1R	colony-stimulating factor 1 receptor
CX3CR1	CX3C chemokine receptor 1
EAE	experimental autoimmune encephalitis
EMP	erythro-myeloid progenitors
FBS	fetal bovine serum
GA	glatiramer acetate
GCV	ganciclovir
GM-CSF	Granulocyte-Macrophage Colony-Stimulating Factor
GTP	guanosine triphosphate
HexB	hexosaminidase subunit beta
HSC	hematopoietic stem cell
HSVTK	herpes simplex virus thymidine kinase
Iba1	ionized calcium-binding adapter molecule 1
IFN-γ	interferon-γ
IL-4	interleukin-4
IL-10	interleukin-10
IL-12	interleukin-12
KO	knockout
Ly6C	lymphocyte antigen 6 complex locus C
LPS	Lipopolysaccharide
LXRs	liver X receptors
MBP	myelin binding protein
MCAO	middle cerebral artery occlusion
M-CSF	Macrophage Colony-Stimulating Factor
MERTK	Mer tyrosine kinase
MS	multiple sclerosis
NAD	nicotinamide adenine dinucleotide
NPCs	neural progenitor cells
Nr4a1	nuclear receptor subfamily 4 group A member 1
P2Y12	P2Y purinoceptor 12
Pbx1	PBX Homeobox 1
RPMI-1640	Roswell Park Memorial Institute-1640
Sall1	SAL-like 1
SCD1	stearoyl-CoA desaturase 1
Siglec-H	sialic acid-binding immunoglobulin-like lectin H
SIRPα	signal regulatory protein α
SIRT1	Sirtuin 1
SLCs	solute carriers
Smad	mothers against decapentaplegic homolog
SR-A	scavenger receptor class A
STAT1	signal transducer and activator of transcription 1
TAMs	tumor-associated macrophages and microglia
TGF-β	transforming growth factor β
TMEM119	transmembrane protein 119
TNF-α	tumor necrosis factor-α
WNV	West Nile virus

## References

[1] Galea I., Bechmann I., Perry V.H. (2007). What is immune privilege (not)?. Trends Immunol..

[2] Niederkorn J.Y. (2006). See no evil, hear no evil, do no evil: The lessons of immune privilege. Nat. Immunol..

[3] Engelhardt B. (2006). Regulation of immune cell entry into the central nervous system. Results Probl. Cell Differ..

[4] Pösel C., Möller K., Boltze J., Wagner D.C., Weise G. (2016). Isolation and Flow Cytometric Analysis of Immune Cells from the Ischemic Mouse Brain. J. Vis. Exp..

[5] Korin B., Ben-Shaanan T.L., Schiller M., Dubovik T., Azulay-Debby H., Boshnak N.T., Koren T., Rolls A. (2017). High-dimensional, single-cell characterization of the brain’s immune compartment. Nat. Neurosci..

[6] Li Q., Barres B.A. (2018). Microglia and macrophages in brain homeostasis and disease. Nat. Rev. Immunol..

[7] Galloway D.A., Phillips A.E.M., Owen D.R.J., Moore C.S. (2019). Phagocytosis in the Brain: Homeostasis and Disease. Front. Immunol..

[8] Kono R., Ikegaya Y., Koyama R. (2021). Phagocytic Glial Cells in Brain Homeostasis. Cells.

[9] Green D.R., Oguin T.H., Martinez J. (2016). The clearance of dying cells: Table for two. Cell Death Differ..

[10] Wolf S.A., Boddeke H.W., Kettenmann H. (2017). Microglia in Physiology and Disease. Annu. Rev. Physiol..

[11] Cavallucci V., D’Amelio M., Cecconi F. (2012). Aβ toxicity in Alzheimer’s disease. Mol. Neurobiol..

[12] Wong Y.C., Krainc D. (2017). α-synuclein toxicity in neurodegeneration: Mechanism and therapeutic strategies. Nat. Med..

[13] Fani Maleki A., Rivest S. (2019). Innate Immune Cells: Monocytes, Monocyte-Derived Macrophages and Microglia as Therapeutic Targets for Alzheimer’s Disease and Multiple Sclerosis. Front. Cell Neurosci..

[14] Alam A., Thelin E.P., Tajsic T., Khan D.Z., Khellaf A., Patani R., Helmy A. (2020). Cellular infiltration in traumatic brain injury. J. Neuroinflamm..

[15] Yamasaki R., Lu H., Butovsky O., Ohno N., Rietsch A.M., Cialic R., Wu P.M., Doykan C.E., Lin J., Cotleur A.C. (2014). Differential roles of microglia and monocytes in the inflamed central nervous system. J. Exp. Med..

[16] Schilling M., Besselmann M., Müller M., Strecker J.K., Ringelstein E.B., Kiefer R. (2005). Predominant phagocytic activity of resident microglia over hematogenous macrophages following transient focal cerebral ischemia: An investigation using green fluorescent protein transgenic bone marrow chimeric mice. Exp. Neurol..

[17] Unger M.S., Schernthaner P., Marschallinger J., Mrowetz H., Aigner L. (2018). Microglia prevent peripheral immune cell invasion and promote an anti-inflammatory environment in the brain of APP-PS1 transgenic mice. J. Neuroinflamm..

[18] Di Liberto G., Pantelyushin S., Kreutzfeldt M., Page N., Musardo S., Coras R., Steinbach K., Vincenti I., Klimek B., Lingner T. (2018). Neurons under T Cell Attack Coordinate Phagocyte-Mediated Synaptic Stripping. Cell.

[19] Prinz M., Erny D., Hagemeyer N. (2017). Ontogeny and homeostasis of CNS myeloid cells. Nat. Immunol..

[20] Prinz M., Masuda T., Wheeler M.A., Quintana F.J. (2021). Microglia and Central Nervous System-Associated Macrophages-From Origin to Disease Modulation. Annu. Rev. Immunol..

[21] Eme-Scolan E., Dando S.J. (2020). Tools and Approaches for Studying Microglia. Front. Immunol..

[22] Spiteri A.G., Wishart C.L., King N.J.C. (2020). Immovable Object Meets Unstoppable Force? Dialogue Between Resident and Peripheral Myeloid Cells in the Inflamed Brain. Front. Immunol..

[23] Grathwohl S.A., Kälin R.E., Bolmont T., Prokop S., Winkelmann G., Kaeser S.A., Odenthal J., Radde R., Eldh T., Gandy S. (2009). Formation and maintenance of Alzheimer’s disease beta-amyloid plaques in the absence of microglia. Nat. Neurosci..

[24] Simard A.R., Soulet D., Gowing G., Julien J.P., Rivest S. (2006). Bone marrow-derived microglia play a critical role in restricting senile plaque formation in Alzheimer’s disease. Neuron.

[25] Hawkes C.A., McLaurin J. (2009). Selective targeting of perivascular macrophages for clearance of beta-amyloid in cerebral amyloid angiopathy. Proc. Natl. Acad. Sci. USA.

[26] Michaud J.P., Bellavance M.A., Préfontaine P., Rivest S. (2013). Real-time in vivo imaging reveals the ability of monocytes to clear vascular amyloid beta. Cell Rep..

[27] Goldmann T., Wieghofer P., Jordão M.J., Prutek F., Hagemeyer N., Frenzel K., Amann L., Staszewski O., Kierdorf K., Krueger M. (2016). Origin, fate and dynamics of macrophages at central nervous system interfaces. Nat. Immunol..

[28] Kucharova K., Stallcup W.B. (2017). Distinct NG2 proteoglycan-dependent roles of resident microglia and bone marrow-derived macrophages during myelin damage and repair. PLoS ONE.

[29] Hutter G., Theruvath J., Graef C.M., Zhang M., Schoen M.K., Manz E.M., Bennett M.L., Olson A., Azad T.D., Sinha R. (2019). Microglia are effector cells of CD47-SIRPα antiphagocytic axis disruption against glioblastoma. Proc. Natl. Acad. Sci. USA.

[30] Greenhalgh A.D., David S. (2014). Differences in the phagocytic response of microglia and peripheral macrophages after spinal cord injury and its effects on cell death. J. Neurosci..

[31] Kamphuis W., Kooijman L., Schetters S., Orre M., Hol E.M. (2016). Transcriptional profiling of CD11c-positive microglia accumulating around amyloid plaques in a mouse model for Alzheimer’s disease. Biochim. Biophys. Acta.

[32] Town T., Laouar Y., Pittenger C., Mori T., Szekely C.A., Tan J., Duman R.S., Flavell R.A. (2008). Blocking TGF-beta-Smad2/3 innate immune signaling mitigates Alzheimer-like pathology. Nat. Med..

[33] Keren-Shaul H., Spinrad A., Weiner A., Matcovitch-Natan O., Dvir-Szternfeld R., Ulland T.K., David E., Baruch K., Lara-Astaiso D., Toth B. (2017). A Unique Microglia Type Associated with Restricting Development of Alzheimer’s Disease. Cell.

[34] Hong S., Beja-Glasser V.F., Nfonoyim B.M., Frouin A., Li S., Ramakrishnan S., Merry K.M., Shi Q., Rosenthal A., Barres B.A. (2016). Complement and microglia mediate early synapse loss in Alzheimer mouse models. Science.

[35] Bennett M.L., Bennett F.C., Liddelow S.A., Ajami B., Zamanian J.L., Fernhoff N.B., Mulinyawe S.B., Bohlen C.J., Adil A., Tucker A. (2016). New tools for studying microglia in the mouse and human CNS. Proc. Natl. Acad. Sci. USA.

[36] Rinner W.A., Bauer J., Schmidts M., Lassmann H., Hickey W.F. (1995). Resident microglia and hematogenous macrophages as phagocytes in adoptively transferred experimental autoimmune encephalomyelitis: An investigation using rat radiation bone marrow chimeras. Glia.

[37] Plemel J.R., Stratton J.A., Michaels N.J., Rawji K.S., Zhang E., Sinha S., Baaklini C.S., Dong Y., Ho M., Thorburn K. (2020). Microglia response following acute demyelination is heterogeneous and limits infiltrating macrophage dispersion. Sci. Adv..

[38] Li S., Hayden E.Y., Garcia V.J., Fuchs D.T., Sheyn J., Daley D.A., Rentsendorj A., Torbati T., Black K.L., Rutishauser U. (2020). Activated Bone Marrow-Derived Macrophages Eradicate Alzheimer’s-Related Aβ. Front. Immunol..

[39] Vasek M.J., Garber C., Dorsey D., Durrant D.M., Bollman B., Soung A., Yu J., Perez-Torres C., Frouin A., Wilton D.K. (2016). A complement-microglial axis drives synapse loss during virus-induced memory impairment. Nature.

[40] Ritzel R.M., Patel A.R., Grenier J.M., Crapser J., Verma R., Jellison E.R., McCullough L.D. (2015). Functional differences between microglia and monocytes after ischemic stroke. J. Neuroinflamm..

[41] Chen Z., Feng X., Herting C.J., Garcia V.A., Nie K., Pong W.W., Rasmussen R., Dwivedi B., Seby S., Wolf S.A. (2017). Cellular and Molecular Identity of Tumor-Associated Macrophages in Glioblastoma. Cancer Res..

[42] Rajan W.D., Wojtas B., Gielniewski B., Gieryng A., Zawadzka M., Kaminska B. (2019). Dissecting functional phenotypes of microglia and macrophages in the rat brain after transient cerebral ischemia. Glia.

[43] Garcia-Bonilla L., Faraco G., Moore J., Murphy M., Racchumi G., Srinivasan J., Brea D., Iadecola C., Anrather J. (2016). Spatio-temporal profile, phenotypic diversity, and fate of recruited monocytes into the post-ischemic brain. J. Neuroinflamm..

[44] Carrillo G.L., Ballard V.A., Glausen T., Boone Z., Teamer J., Hinkson C.L., Wohlfert E.A., Blader I.J., Fox M.A. (2020). Toxoplasma infection induces microglia-neuron contact and the loss of perisomatic inhibitory synapses. Glia.

[45] Yona S., Kim K.W., Wolf Y., Mildner A., Varol D., Breker M., Strauss-Ayali D., Viukov S., Guilliams M., Misharin A. (2013). Fate mapping reveals origins and dynamics of monocytes and tissue macrophages under homeostasis. Immunity.

[46] Mildner A., Schönheit J., Giladi A., David E., Lara-Astiaso D., Lorenzo-Vivas E., Paul F., Chappell-Maor L., Priller J., Leutz A. (2017). Genomic Characterization of Murine Monocytes Reveals C/EBPβ Transcription Factor Dependence of Ly6C. Immunity.

[47] Holtman I.R., Raj D.D., Miller J.A., Schaafsma W., Yin Z., Brouwer N., Wes P.D., Möller T., Orre M., Kamphuis W. (2015). Induction of a common microglia gene expression signature by aging and neurodegenerative conditions: A co-expression meta-analysis. Acta Neuropathol. Commun..

[48] Butovsky O., Weiner H.L. (2018). Microglial signatures and their role in health and disease. Nat. Rev. Neurosci.

[49] Chen H.R., Sun Y.Y., Chen C.W., Kuo Y.M., Kuan I.S., Tiger Li Z.R., Short-Miller J.C., Smucker M.R., Kuan C.Y. (2020). Fate mapping via CCR2-CreER mice reveals monocyte-to-microglia transition in development and neonatal stroke. Sci. Adv..

[50] Masuda T., Amann L., Sankowski R., Staszewski O., Lenz M., Errico P.D., Snaidero N., Costa Jordão M.J., Böttcher C., Kierdorf K. (2020). Novel Hexb-based tools for studying microglia in the CNS. Nat. Immunol..

[51] Cunningham C.L., Martínez-Cerdeño V., Noctor S.C. (2013). Microglia regulate the number of neural precursor cells in the developing cerebral cortex. J. Neurosci..

[52] Perez-Pouchoulen M., VanRyzin J.W., McCarthy M.M. (2015). Morphological and Phagocytic Profile of Microglia in the Developing Rat Cerebellum. eNeuro.

[53] VanRyzin J.W., Marquardt A.E., Argue K.J., Vecchiarelli H.A., Ashton S.E., Arambula S.E., Hill M.N., McCarthy M.M. (2019). Microglial Phagocytosis of Newborn Cells Is Induced by Endocannabinoids and Sculpts Sex Differences in Juvenile Rat Social Play. Neuron.

[54] Fourgeaud L., Través P.G., Tufail Y., Leal-Bailey H., Lew E.D., Burrola P.G., Callaway P., Zagórska A., Rothlin C.V., Nimmerjahn A. (2016). TAM receptors regulate multiple features of microglial physiology. Nature.

[55] Sierra A., Abiega O., Shahraz A., Neumann H. (2013). Janus-faced microglia: Beneficial and detrimental consequences of microglial phagocytosis. Front. Cell Neurosci..

[56] Sierra A., Encinas J.M., Deudero J.J., Chancey J.H., Enikolopov G., Overstreet-Wadiche L.S., Tsirka S.E., Maletic-Savatic M. (2010). Microglia shape adult hippocampal neurogenesis through apoptosis-coupled phagocytosis. Cell Stem Cell.

[57] Mazaheri F., Breus O., Durdu S., Haas P., Wittbrodt J., Gilmour D., Peri F. (2014). Distinct roles for BAI1 and TIM-4 in the engulfment of dying neurons by microglia. Nat. Commun..

[58] Marín-Teva J.L., Dusart I., Colin C., Gervais A., van Rooijen N., Mallat M. (2004). Microglia promote the death of developing Purkinje cells. Neuron.

[59] Li Q., Cheng Z., Zhou L., Darmanis S., Neff N.F., Okamoto J., Gulati G., Bennett M.L., Sun L.O., Clarke L.E. (2019). Developmental Heterogeneity of Microglia and Brain Myeloid Cells Revealed by Deep Single-Cell RNA Sequencing. Neuron.

[60] Safaiyan S., Kannaiyan N., Snaidero N., Brioschi S., Biber K., Yona S., Edinger A.L., Jung S., Rossner M.J., Simons M. (2016). Age-related myelin degradation burdens the clearance function of microglia during aging. Nat. Neurosci..

[61] Hill R.A., Li A.M., Grutzendler J. (2018). Lifelong cortical myelin plasticity and age-related degeneration in the live mammalian brain. Nat. Neurosci..

[62] Courchesne E., Mouton P.R., Calhoun M.E., Semendeferi K., Ahrens-Barbeau C., Hallet M.J., Barnes C.C., Pierce K. (2011). Neuron number and size in prefrontal cortex of children with autism. JAMA.

[63] Paolicelli R.C., Bolasco G., Pagani F., Maggi L., Scianni M., Panzanelli P., Giustetto M., Ferreira T.A., Guiducci E., Dumas L. (2011). Synaptic pruning by microglia is necessary for normal brain development. Science.

[64] Schafer D.P., Lehrman E.K., Kautzman A.G., Koyama R., Mardinly A.R., Yamasaki R., Ransohoff R.M., Greenberg M.E., Barres B.A., Stevens B. (2012). Microglia sculpt postnatal neural circuits in an activity and complement-dependent manner. Neuron.

[65] Weinhard L., di Bartolomei G., Bolasco G., Machado P., Schieber N.L., Neniskyte U., Exiga M., Vadisiute A., Raggioli A., Schertel A. (2018). Microglia remodel synapses by presynaptic trogocytosis and spine head filopodia induction. Nat. Commun..

[66] Kim H.J., Cho M.H., Shim W.H., Kim J.K., Jeon E.Y., Kim D.H., Yoon S.Y. (2017). Deficient autophagy in microglia impairs synaptic pruning and causes social behavioral defects. Mol. Psychiatry.

[67] Filipello F., Morini R., Corradini I., Zerbi V., Canzi A., Michalski B., Erreni M., Markicevic M., Starvaggi-Cucuzza C., Otero K. (2018). The Microglial Innate Immune Receptor TREM2 Is Required for Synapse Elimination and Normal Brain Connectivity. Immunity.

[68] Chu Y., Jin X., Parada I., Pesic A., Stevens B., Barres B., Prince D.A. (2010). Enhanced synaptic connectivity and epilepsy in C1q knockout mice. Proc. Natl. Acad. Sci. USA.

[69] Andoh M., Shibata K., Okamoto K., Onodera J., Morishita K., Miura Y., Ikegaya Y., Koyama R. (2019). Exercise Reverses Behavioral and Synaptic Abnormalities after Maternal Inflammation. Cell Rep..

[70] Sipe G.O., Lowery R.L., Tremblay M., Kelly E.A., Lamantia C.E., Majewska A.K. (2016). Microglial P2Y12 is necessary for synaptic plasticity in mouse visual cortex. Nat. Commun..

[71] Wang C., Yue H., Hu Z., Shen Y., Ma J., Li J., Wang X.D., Wang L., Sun B., Shi P. (2020). Microglia mediate forgetting via complement-dependent synaptic elimination. Science.

[72] Andoh M., Koyama R. (2021). Microglia regulate synaptic development and plasticity. Dev. Neurobiol..

[73] Peters A., Moss M.B., Sethares C. (2000). Effects of aging on myelinated nerve fibers in monkey primary visual cortex. J. Comp. Neurol..

[74] Jeon S.B., Yoon H.J., Park S.H., Kim I.H., Park E.J. (2008). Sulfatide, a major lipid component of myelin sheath, activates inflammatory responses as an endogenous stimulator in brain-resident immune cells. J. Immunol..

[75] Lampron A., Larochelle A., Laflamme N., Préfontaine P., Plante M.M., Sánchez M.G., Yong V.W., Stys P.K., Tremblay M., Rivest S. (2015). Inefficient clearance of myelin debris by microglia impairs remyelinating processes. J. Exp. Med..

[76] Mosley K., Cuzner M.L. (1996). Receptor-mediated phagocytosis of myelin by macrophages and microglia: Effect of opsonization and receptor blocking agents. Neurochem. Res..

[77] Durafourt B.A., Moore C.S., Zammit D.A., Johnson T.A., Zaguia F., Guiot M.C., Bar-Or A., Antel J.P. (2012). Comparison of polarization properties of human adult microglia and blood-derived macrophages. Glia.

[78] Fu R., Shen Q., Xu P., Luo J.J., Tang Y. (2014). Phagocytosis of microglia in the central nervous system diseases. Mol. Neurobiol..

[79] Palle P., Monaghan K.L., Milne S.M., Wan E.C.K. (2017). Cytokine Signaling in Multiple Sclerosis and Its Therapeutic Applications. Med. Sci..

[80] Smith M.E. (1999). Phagocytosis of myelin in demyelinative disease: A review. Neurochem. Res..

[81] Greenhalgh A.D., Zarruk J.G., Healy L.M., Baskar Jesudasan S.J., Jhelum P., Salmon C.K., Formanek A., Russo M.V., Antel J.P., McGavern D.B. (2018). Peripherally derived macrophages modulate microglial function to reduce inflammation after CNS injury. PLoS Biol..

[82] Poon I.K., Lucas C.D., Rossi A.G., Ravichandran K.S. (2014). Apoptotic cell clearance: Basic biology and therapeutic potential. Nat. Rev. Immunol..

[83] Gholamin S., Mitra S.S., Feroze A.H., Liu J., Kahn S.A., Zhang M., Esparza R., Richard C., Ramaswamy V., Remke M. (2017). Disrupting the CD47-SIRPα anti-phagocytic axis by a humanized anti-CD47 antibody is an efficacious treatment for malignant pediatric brain tumors. Sci. Transl. Med..

[84] Haass C., Selkoe D.J. (2007). Soluble protein oligomers in neurodegeneration: Lessons from the Alzheimer’s amyloid beta-peptide. Nat. Rev. Mol. Cell Biol..

[85] Jack C.R., Knopman D.S., Jagust W.J., Petersen R.C., Weiner M.W., Aisen P.S., Shaw L.M., Vemuri P., Wiste H.J., Weigand S.D. (2013). Tracking pathophysiological processes in Alzheimer’s disease: An updated hypothetical model of dynamic biomarkers. Lancet Neurol..

[86] Lai A.Y., McLaurin J. (2012). Clearance of amyloid-β peptides by microglia and macrophages: The issue of what, when and where. Future Neurol..

[87] Mandrekar S., Jiang Q., Lee C.Y., Koenigsknecht-Talboo J., Holtzman D.M., Landreth G.E. (2009). Microglia mediate the clearance of soluble Abeta through fluid phase macropinocytosis. J. Neurosci..

[88] Liu Z., Condello C., Schain A., Harb R., Grutzendler J. (2010). CX3CR1 in microglia regulates brain amyloid deposition through selective protofibrillar amyloid-β phagocytosis. J. Neurosci..

[89] Frackowiak J., Wisniewski H.M., Wegiel J., Merz G.S., Iqbal K., Wang K.C. (1992). Ultrastructure of the microglia that phagocytose amyloid and the microglia that produce beta-amyloid fibrils. Acta Neuropathol..

[90] Huang Y., Happonen K.E., Burrola P.G., O’Connor C., Hah N., Huang L., Nimmerjahn A., Lemke G. (2021). Microglia use TAM receptors to detect and engulf amyloid β plaques. Nat. Immunol..

[91] Fiala M., Lin J., Ringman J., Kermani-Arab V., Tsao G., Patel A., Lossinsky A.S., Graves M.C., Gustavson A., Sayre J. (2005). Ineffective phagocytosis of amyloid-beta by macrophages of Alzheimer’s disease patients. J. Alzheimer’s Dis..

[92] Ajami B., Bennett J.L., Krieger C., Tetzlaff W., Rossi F.M. (2007). Local self-renewal can sustain CNS microglia maintenance and function throughout adult life. Nat. Neurosci..

[93] Mildner A., Schmidt H., Nitsche M., Merkler D., Hanisch U.K., Mack M., Heikenwalder M., Brück W., Priller J., Prinz M. (2007). Microglia in the adult brain arise from Ly-6ChiCCR2+ monocytes only under defined host conditions. Nat. Neurosci..

[94] Condello C., Yuan P., Schain A., Grutzendler J. (2015). Microglia constitute a barrier that prevents neurotoxic protofibrillar Aβ42 hotspots around plaques. Nat. Commun..

[95] Tichauer J.E., von Bernhardi R. (2012). Transforming growth factor-β stimulates β amyloid uptake by microglia through Smad3-dependent mechanisms. J. Neurosci. Res..

[96] Colangelo V., Schurr J., Ball M.J., Pelaez R.P., Bazan N.G., Lukiw W.J. (2002). Gene expression profiling of 12633 genes in Alzheimer hippocampal CA1: Transcription and neurotrophic factor down-regulation and up-regulation of apoptotic and pro-inflammatory signaling. J. Neurosci Res..

[97] Tichauer J.E., Flores B., Soler B., Eugenín-von Bernhardi L., Ramírez G., von Bernhardi R. (2014). Age-dependent changes on TGFβ1 Smad3 pathway modify the pattern of microglial cell activation. Brain Behav. Immun..

[98] Von Bernhardi R., Cornejo F., Parada G.E., Eugenín J. (2015). Role of TGFβ signaling in the pathogenesis of Alzheimer’s disease. Front. Cell Neurosci..

[99] Mucke L., Selkoe D.J. (2012). Neurotoxicity of amyloid β-protein: Synaptic and network dysfunction. Cold Spring Harb. Perspect. Med..

[100] Hong S., Ostaszewski B.L., Yang T., O’Malley T.T., Jin M., Yanagisawa K., Li S., Bartels T., Selkoe D.J. (2014). Soluble Aβ oligomers are rapidly sequestered from brain ISF in vivo and bind GM1 ganglioside on cellular membranes. Neuron.

[101] Koronyo Y., Salumbides B.C., Sheyn J., Pelissier L., Li S., Ljubimov V., Moyseyev M., Daley D., Fuchs D.T., Pham M. (2015). Therapeutic effects of glatiramer acetate and grafted CD115⁺ monocytes in a mouse model of Alzheimer’s disease. Brain.

[102] Rentsendorj A., Sheyn J., Fuchs D.T., Daley D., Salumbides B.C., Schubloom H.E., Hart N.J., Li S., Hayden E.Y., Teplow D.B. (2018). A novel role for osteopontin in macrophage-mediated amyloid-β clearance in Alzheimer’s models. Brain Behav. Immun..

[103] Chan J.L., Reeves T.M., Phillips L.L. (2014). Osteopontin expression in acute immune response mediates hippocampal synaptogenesis and adaptive outcome following cortical brain injury. Exp. Neurol..

[104] Wang Y., Ulland T.K., Ulrich J.D., Song W., Tzaferis J.A., Hole J.T., Yuan P., Mahan T.E., Shi Y., Gilfillan S. (2016). TREM2-mediated early microglial response limits diffusion and toxicity of amyloid plaques. J. Exp. Med..

[105] Li Y., Severance E.G., Viscidi R.P., Yolken R.H., Xiao J. (2019). Persistent Toxoplasma Infection of the Brain Induced Neurodegeneration Associated with Activation of Complement and Microglia. Infect. Immun..

[106] Xiao J., Li Y., Gressitt K.L., He H., Kannan G., Schultz T.L., Svezhova N., Carruthers V.B., Pletnikov M.V., Yolken R.H. (2016). Cerebral complement C1q activation in chronic Toxoplasma infection. Brain Behav. Immun..

[107] Saederup N., Cardona A.E., Croft K., Mizutani M., Cotleur A.C., Tsou C.L., Ransohoff R.M., Charo I.F. (2017). Correction: Selective Chemokine Receptor Usage by Central Nervous System Myeloid Cells in CCR2-Red Fluorescent Protein Knock-In Mice. PLoS ONE.

[108] Kourtzelis I., Hajishengallis G., Chavakis T. (2020). Phagocytosis of Apoptotic Cells in Resolution of Inflammation. Front. Immunol..

[109] Kim S., Elkon K.B., Ma X. (2004). Transcriptional suppression of interleukin-12 gene expression following phagocytosis of apoptotic cells. Immunity.

[110] Bogie J.F., Timmermans S., Huynh-Thu V.A., Irrthum A., Smeets H.J., Gustafsson J., Steffensen K.R., Mulder M., Stinissen P., Hellings N. (2012). Myelin-derived lipids modulate macrophage activity by liver X receptor activation. PLoS ONE.

[111] Bogie J.F., Jorissen W., Mailleux J., Nijland P.G., Zelcer N., Vanmierlo T., Van Horssen J., Stinissen P., Hellings N., Hendriks J.J. (2013). Myelin alters the inflammatory phenotype of macrophages by activating PPARs. Acta Neuropathol. Commun..

[112] Bogie J.F.J., Grajchen E., Wouters E., Corrales A.G., Dierckx T., Vanherle S., Mailleux J., Gervois P., Wolfs E., Dehairs J. (2020). Stearoyl-CoA desaturase-1 impairs the reparative properties of macrophages and microglia in the brain. J. Exp. Med..

[113] Morioka S., Perry J.S.A., Raymond M.H., Medina C.B., Zhu Y., Zhao L., Serbulea V., Onengut-Gumuscu S., Leitinger N., Kucenas S. (2018). Efferocytosis induces a novel SLC program to promote glucose uptake and lactate release. Nature.

[114] Zhang S., Weinberg S., DeBerge M., Gainullina A., Schipma M., Kinchen J.M., Ben-Sahra I., Gius D.R., Yvan-Charvet L., Chandel N.S. (2019). Efferocytosis Fuels Requirements of Fatty Acid Oxidation and the Electron Transport Chain to Polarize Macrophages for Tissue Repair. Cell Metab..

[115] Saftig P., Klumperman J. (2009). Lysosome biogenesis and lysosomal membrane proteins: Trafficking meets function. Nat. Rev. Mol. Cell Biol..

[116] Andoh M., Koyama R. (2021). Assessing Microglial Dynamics by Live Imaging. Front. Immunol..

[117] Yurdagul A., Subramanian M., Wang X., Crown S.B., Ilkayeva O.R., Darville L., Kolluru G.K., Rymond C.C., Gerlach B.D., Zheng Z. (2020). Macrophage Metabolism of Apoptotic Cell-Derived Arginine Promotes Continual Efferocytosis and Resolution of Injury. Cell Metab..

[118] Grajchen E., Wouters E., van de Haterd B., Haidar M., Hardonnière K., Dierckx T., Van Broeckhoven J., Erens C., Hendrix S., Kerdine-Römer S. (2020). CD36-mediated uptake of myelin debris by macrophages and microglia reduces neuroinflammation. J. Neuroinflamm..

[119] Ralston K.S., Solga M.D., Mackey-Lawrence N.M., Somlata, Bhattacharya A., Petri W.A. (2014). Trogocytosis by Entamoeba histolytica contributes to cell killing and tissue invasion. Nature.

[120] Miller H.W., Suleiman R.L., Ralston K.S. (2019). Trogocytosis by Entamoeba histolytica Mediates Acquisition and Display of Human Cell Membrane Proteins and Evasion of Lysis by Human Serum. MBio.

[121] Casano A.M., Albert M., Peri F. (2016). Developmental Apoptosis Mediates Entry and Positioning of Microglia in the Zebrafish Brain. Cell Rep..

[122] Xu J., Wang T., Wu Y., Jin W., Wen Z. (2016). Microglia Colonization of Developing Zebrafish Midbrain Is Promoted by Apoptotic Neuron and Lysophosphatidylcholine. Dev. Cell.

[123] Schetters S.T.T., Gomez-Nicola D., Garcia-Vallejo J.J., Van Kooyk Y. (2017). Neuroinflammation: Microglia and T Cells Get Ready to Tango. Front. Immunol..

[124] Mundt S., Mrdjen D., Utz S.G., Greter M., Schreiner B., Becher B. (2019). Conventional DCs sample and present myelin antigens in the healthy CNS and allow parenchymal T cell entry to initiate neuroinflammation. Sci. Immunol..

[125] Asai H., Ikezu S., Tsunoda S., Medalla M., Luebke J., Haydar T., Wolozin B., Butovsky O., Kügler S., Ikezu T. (2015). Depletion of microglia and inhibition of exosome synthesis halt tau propagation. Nat. Neurosci..

[126] Zhu K., Pieber M., Han J., Blomgren K., Zhang X.M., Harris R.A., Lund H. (2020). Absence of microglia or presence of peripherally-derived macrophages does not affect tau pathology in young or old hTau mice. Glia.

[127] Yuan D., Zhao Y., Banks W.A., Bullock K.M., Haney M., Batrakova E., Kabanov A.V. (2017). Macrophage exosomes as natural nanocarriers for protein delivery to inflamed brain. Biomaterials.

[128] Dou Y., Wu H.J., Li H.Q., Qin S., Wang Y.E., Li J., Lou H.F., Chen Z., Li X.M., Luo Q.M. (2012). Microglial migration mediated by ATP-induced ATP release from lysosomes. Cell Res..

[129] Pasqual G., Chudnovskiy A., Tas J.M.J., Agudelo M., Schweitzer L.D., Cui A., Hacohen N., Victora G.D. (2018). Monitoring T cell-dendritic cell interactions in vivo by intercellular enzymatic labelling. Nature.

[130] Giladi A., Cohen M., Medaglia C., Baran Y., Li B., Zada M., Bost P., Blecher-Gonen R., Salame T.M., Mayer J.U. (2020). Dissecting cellular crosstalk by sequencing physically interacting cells. Nat. Biotechnol..

[131] Clark I.C., Gutiérrez-Vázquez C., Wheeler M.A., Li Z., Rothhammer V., Linnerbauer M., Sanmarco L.M., Guo L., Blain M., Zandee S.E.J. (2021). Barcoded viral tracing of single-cell interactions in central nervous system inflammation. Science.

[132] Muffat J., Li Y., Yuan B., Mitalipova M., Omer A., Corcoran S., Bakiasi G., Tsai L.H., Aubourg P., Ransohoff R.M. (2016). Efficient derivation of microglia-like cells from human pluripotent stem cells. Nat. Med..

[133] Abud E.M., Ramirez R.N., Martinez E.S., Healy L.M., Nguyen C.H.H., Newman S.A., Yeromin A.V., Scarfone V.M., Marsh S.E., Fimbres C. (2017). iPSC-Derived Human Microglia-like Cells to Study Neurological Diseases. Neuron.

[134] Douvaras P., Sun B., Wang M., Kruglikov I., Lallos G., Zimmer M., Terrenoire C., Zhang B., Gandy S., Schadt E. (2017). Directed Differentiation of Human Pluripotent Stem Cells to Microglia. Stem Cell Rep..

[135] Haenseler W., Sansom S.N., Buchrieser J., Newey S.E., Moore C.S., Nicholls F.J., Chintawar S., Schnell C., Antel J.P., Allen N.D. (2017). A Highly Efficient Human Pluripotent Stem Cell Microglia Model Displays a Neuronal-Co-culture-Specific Expression Profile and Inflammatory Response. Stem Cell Rep..

[136] Pandya H., Shen M.J., Ichikawa D.M., Sedlock A.B., Choi Y., Johnson K.R., Kim G., Brown M.A., Elkahloun A.G., Maric D. (2017). Differentiation of human and murine induced pluripotent stem cells to microglia-like cells. Nat. Neurosci..

[137] Takata K., Kozaki T., Lee C.Z.W., Thion M.S., Otsuka M., Lim S., Utami K.H., Fidan K., Park D.S., Malleret B. (2017). Induced-Pluripotent-Stem-Cell-Derived Primitive Macrophages Provide a Platform for Modeling Tissue-Resident Macrophage Differentiation and Function. Immunity.

[138] Ohgidani M., Kato T.A., Setoyama D., Sagata N., Hashimoto R., Shigenobu K., Yoshida T., Hayakawa K., Shimokawa N., Miura D. (2014). Direct induction of ramified microglia-like cells from human monocytes: Dynamic microglial dysfunction in Nasu-Hakola disease. Sci. Rep..

[139] Sellgren C.M., Sheridan S.D., Gracias J., Xuan D., Fu T., Perlis R.H. (2017). Patient-specific models of microglia-mediated engulfment of synapses and neural progenitors. Mol. Psychiatry.

